# Comparison of Policosanol Profiles of the Sprouts of Wheat Mutant Lines and the Effect of Differential LED Lights on Selected Lines

**DOI:** 10.3390/plants12193377

**Published:** 2023-09-25

**Authors:** Ah-Reum Han, Euna Choi, Jisu Park, Sang-Hee Jo, Min Jeong Hong, Jin-Baek Kim, Ga-Hee Ryoo, Chang Hyun Jin

**Affiliations:** Advanced Radiation Technology Institute, Korea Atomic Energy Research Institute, Jeongeup-si 56212, Republic of Korea; euna3388@kaeri.re.kr (E.C.); parkjs94@kaeri.re.kr (J.P.); shjo@kaeri.re.kr (S.-H.J.); hongmj@kaeri.re.kr (M.J.H.); jbkim74@kaeri.re.kr (J.-B.K.); ghryoo@kaeri.re.kr (G.-H.R.); chjin@kaeri.re.kr (C.H.J.)

**Keywords:** wheat, *Triticum aestivum*, policosanol, GC-MS, LED light

## Abstract

Policosanols (PCs) are long-chain linear aliphatic alcohols that are present in the primary leaves of cereal crops, such as barley and wheat, sugar cane wax, and beeswax. PCs have been used as a nutraceutical for improving hyperlipidemia and hypercholesterolemia. However, the PC content in mutant wheat lines has not been investigated. To select highly functional wheat sprouts with a high content of PCs in wheat mutant lines developed via gamma-irradiated mutation breeding, we cultivated the sprouts of wheat mutant lines in a growth chamber with white LED light (6000 K) and analyzed the PC content in these samples using GC-MS. We studied the PC content in 91 wheat sprout samples: the original variety (Woori-mil × D-7; WS01), commercially available cv. Geumgang (WS87) and cv. Cheongwoo (WS91), and mutant lines (WS02–WS86 and WS88–WS90) developed from WS01 and WS87. Compared to WS01, 18 mutant lines exhibited a high total PC content (506.08–873.24 mg/100 g dry weight). Among them, the top 10 mutant lines were evaluated for their PC production after cultivating under blue (440 nm), green (520 nm), and red (660 nm) LED light irradiation; however, these colored LED lights reduced the total PC production by 35.8–49.7%, suggesting that the cultivation with white LED lights was more efficient in promoting PCs’ yield, compared to different LED lights. Therefore, our findings show the potential of radiation-bred wheat varieties as functional foods against hyperlipidemia and obesity and the optimal light conditions for high PC production.

## 1. Introduction

Wheat (*Triticum aestivum* L.) is a staple crop that ranks third in global grain production and serves as a source of energy and an important nutrient in the human diet [1]. Wheat contains not only nutrients, including vitamins, proteins, minerals, and dietary fiber, but also health-promoting compounds, such as flavonoids, lignans, phenolic acids, alkylresorcinols, benzoxazinoids, steroids, sphingolipids, fatty acids, and glycolipids [2]. Wheat flour is a staple food worldwide. However, wheat sprouts have recently attracted considerable attention as a functional food [3], as sprouts can improve their nutritional value and produce more health-promoting compounds than grains [4]. Flavonoid C-glycosides and policosanols (PCs) are major bioactive components in wheat sprouts [5,6,7]. Wheat sprout extract has been reported to exhibit antioxidant [8], anticancer [9], antimutagenic [10], anti-osteoporotic [11], hepatoprotective [12], and hyperlipidemic activities [6].

PCs are mixtures of long-chain aliphatic primary alcohols derived from plant wax [13,14] or insect wax [15,16]. Previous studies have demonstrated that PCs can inhibit 3-hydroxy-3-methylglutaryl-CoA (HMG-CoA) reductase activity by adenosine 5′-monophosphate-activated protein kinase (AMPK) phosphorylation, which inhibits cholesterol synthesis [17], and clinical studies on PCs have shown that it has lipid-lowering, LDL-cholesterol-lowering, and HDL-cholesterol-increasing effects [18]. However, results from several randomized controlled trials conducted in Europe and the United States provided insufficient evidence of PCs’ significant effect on plasma cholesterol levels [19]. For this reason, the European Food Safety Authority has rejected claims about the beneficial effects of PCs [20]. Research on the health beneficial effects of PCs has been continuously conducted, including a study on the in vivo mechanism of policosanols on hypercholesterolemia induced by a high-fat and high-cholesterol diet in rats [21]. In addition, PCs attenuate hepatic lipid accumulation [22] and vascular calcification [23] and stimulate osteoblast differentiation [24]. These effects have been attributed to the inhibition of sterol biosynthesis via the regulation of AMPK [21,22,23,24]. Recently, several in vivo studies have also shown that PCs have protective effects against Alzheimer’s [14,15] and Parkinson’s diseases [16]. Hence, researchers investigated the composition and content of PCs in cereal sprouts, including wheat, barley, and oats [6,7,25,26]. However, the PC content in wheat mutant lines developed via gamma-irradiated mutation breeding has not been studied thus far.

Environmental factors such as artificial light, water, temperature, and carbon dioxide influence the increased production of various secondary metabolites in plants [27,28,29]. Several studies of sprout cultivation in controlled environments using diverse wavelengths of light emitting diodes (LEDs), including red, blue, and green, and their combinations, have been reported to enhance the accumulation of health-promoting compounds and biomass production [7,30,31,32]. The environment controlled by different wavelengths, intensities, and cycles of LED light can modify the photosynthetic process in plants [33]. Photomorphogenic responses are regulated by plant photoreceptors such as phytochromes and cryptochromes [34]. Phytochromes regulate plants’ physiological responses and synthesize phytochemicals such as phenolic substances [35]. Cryptochromes regulate biomass production and the biosynthesis of anthocyanins, carotenoids, and chlorophyll [36,37]. PCs, very long-chain fatty acid alcohols, were present in wheat leaf cuticle wax, and fatty acyl coenzyme A reductase (FAR) is known to be involved in the biosynthesis of long-chain primary alcohols [38]. In diploid *Aegilops tauschii*, the D-genome donor for hexaploid wheat (*T. aestivum*), five FARs are found to be responsible for primary alcohol (C16–C28) biosynthesis [39]. Recently, the policosanol content and the expression pattern of its biosynthesis-related genes in sprouts of barley and wheat cultivated under LED light irradiation were studied [7].

In this study, the composition and content of PCs in the original variety and its mutant lines, cultivated in an environmentally controlled growth chamber, were determined using gas chromatography–mass spectrometry (GC-MS). The individual and total PC contents in these samples were compared to the selected mutant lines with a high PC content. In addition, we evaluated the effects of different LED light sources on the production of PCs and the growth quality of the 10 selected mutant lines under controlled conditions.

## 2. Results and Discussion

### 2.1. Identification of Policosanols in the Wheat Sprout Samples Using GC–MS Analysis

The PCs in the *n*-hexane extracts of wheat sprout samples were identified via GC-MS using the trimethylsilane (TMS) derivatives of PC standards. The mass spectra of the respective PC-TMS derivatives showed ion peaks of [M−15]^+^ fragmentation, indicating the loss of a -CH_3_ group, and exhibited characteristic fragment ions at *m*/*z* 103 [CH_2_OSi-(CH_3_)_3_^+^], *m*/*z* 75 [HO–Si(CH_3_)_2_^+^], and *m*/*z* 15 [C_4_H_9_^+^], indicating TMS alcohols (Figure S1). The GC-MS retention times (*t*_R_) for the PC-TMS derivatives are listed in Table 1. Calibration curves of the PC-TMS derivatives were prepared for quantitation using 2, 5, 10, and 20 μg/mL of eicosanol (C20-OH), heneicosanol (C21-OH), docosanol (C22-OH), tricosanol (C23-OH), tetracosanol (C24-OH), and hexacosanol (C26-OH), as well as 5, 10, 20, and 50 μg/mL of heptacosanol (C27-OH), octacosanol (C28-OH), and triacontanol (C30-OH). The second-order polynomial equations for these compounds were calculated (Section 4.2.) The calibration curves were curvilinear with high correlation coefficients (*R*^2^ = 0.996−1.000).

### 2.2. Comparison of Policosanol Contents in Different Wheat Sprout Samples

Several studies have analyzed the PC contents of different varieties of wheat and barley sprouts cultivated at different growth times and under different LED light conditions [6,7,25,26]. However, the PC contents of wheat mutant lines developed via gamma-irradiated mutation breeding have not yet been evaluated. Initially, we confirmed the production of PCs in the wheat sprout samples cultivated in a growth chamber with white LED light (6000 K). This analytical method was then used to investigate the contents of the PC-TMS derivatives in wheat sprout samples. The individual and total PCs in the wheat sprout samples differed considerably (Table 2). Octacosanol (C28-OH) was the most abundant PC in the wheat sprout samples, followed by triacontanol (C30-OH) and hexacosanol (C26-OH). Moreover, the contents of heptacosanol (C27-OH), hexacosanol (C26-OH), tetracosanol (C24-OH), tricosanol (C23-OH), and docosanol (C22-OH) were lower than those of the three aforementioned compounds. Notably, eicosanol (C21-OH) and heneicosanol (C20-OH) were not detected in the GC-MS analysis of the wheat sprout samples (Figure S2).

Among the 85 sprout samples of the wheat mutant lines (WS02–WS86) derived from the original variety (WS01), 19 mutant lines showed a higher total PC content than that of WS01. WS74 exhibited the highest total PC content, followed by WS37, WS69, WS72, WS76, WS57, WS81, WS78, WS49, WS75, WS31, WS79, WS51, WS46, WS63, WS48, WS70, WS66, and WS40. Octacosanol (C28-OH) was the most abundant PC in all the mutant lines, accounting for approximately 75% of the average total PC content. Triacontanol (C30-OH) and hexacosanol (C26-OH) were the second and third major components, accounting for ~13.5% and ~6.5% of the average total PC content, respectively. The remaining PCs accounted for <2% of the average total PC content, indicating that they are very minor components.

**Figure 1:**
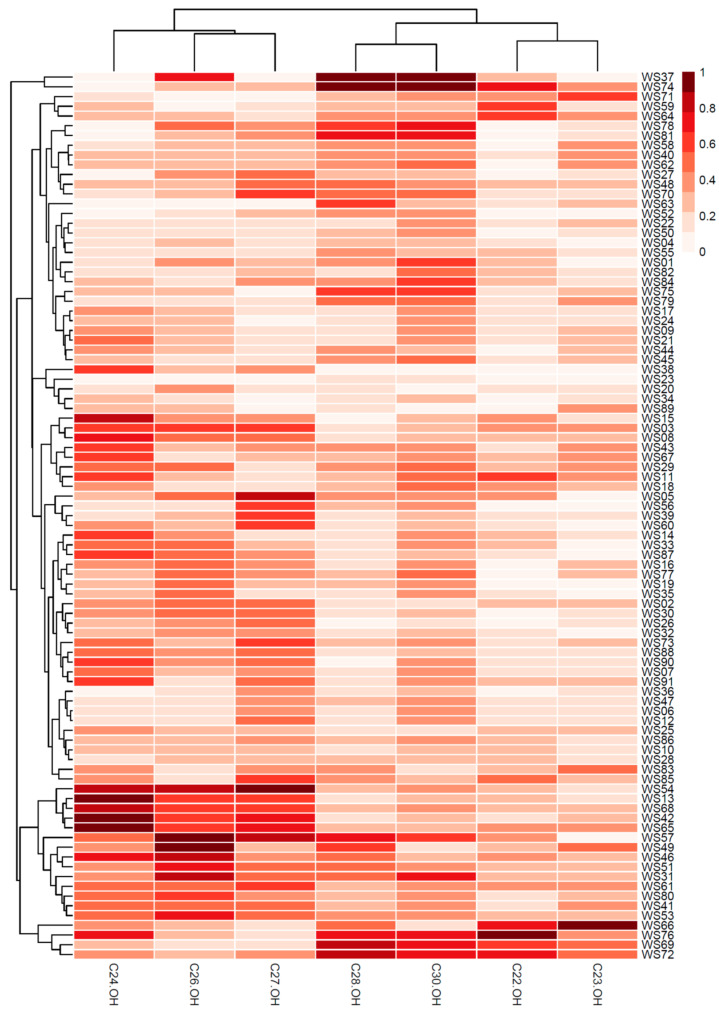
Hierarchical clustering analysis (HCA) with a heatmap for the wheat sprout samples.

The concentration values of the PCs of the 91 wheat sprout samples were exported for hierarchical clustering analysis. HCA with a heatmap was performed to determine the sample classification and to highlight variations in wheat sprout samples’ PC composition (Figure 1). Among the improved 19 mutant lines, WS37 and WS74 were clustered at the top with the highest quantification of octacosanol (C28-OH) and triacontanol (C30-OH). Similar to these, eight mutant lines, WS78, WS81, WS40, WS48, WS70, WS63, WS75, and WS79, showed a relatively high quantification of octacosanol (C28-OH) and triacontanol (C30-OH) compared to the other components and were clustered together. Four mutant lines, WS66, WS76, WS69, and WS72, with a high quantification of octacosanol (C28-OH) and triacontanol (C30-OH) as well as a high quantification of docosanol (C22-OH) and tricosanol (C23-OH) were clustered together. Five mutant lines, WS57, WS49, WS46, WS51, and WS31, with a high quantification of hexacosanol (C26-OH), heptacosanol (C27-OH), octacosanol (C28-OH) were clustered together. These two clusters were merged into one supergroup.

**Figure 2:**
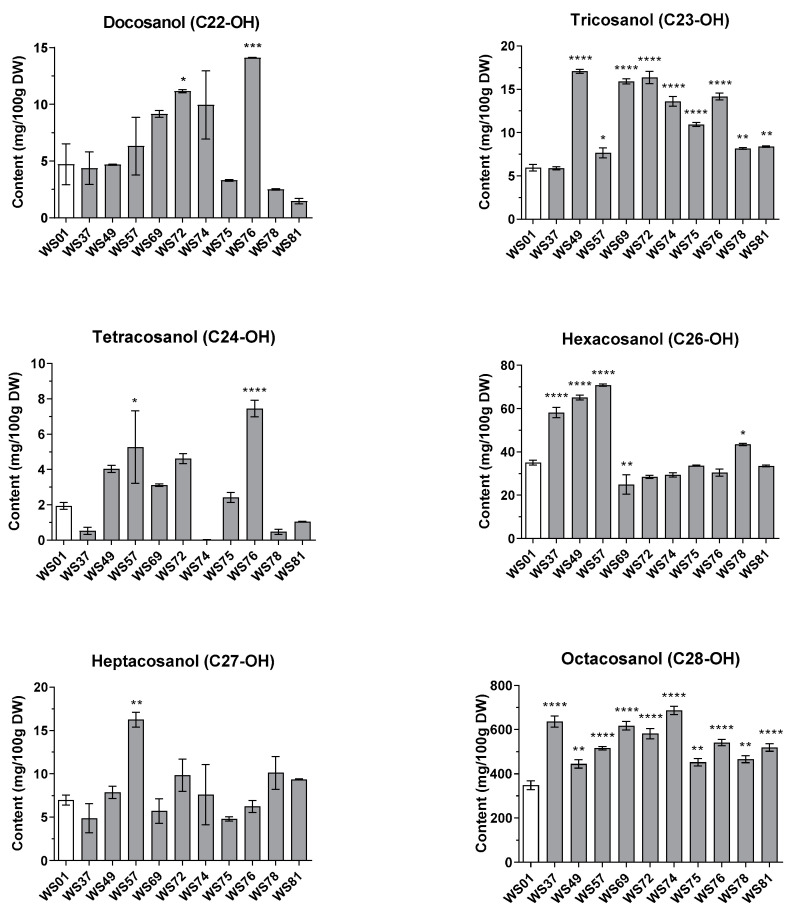

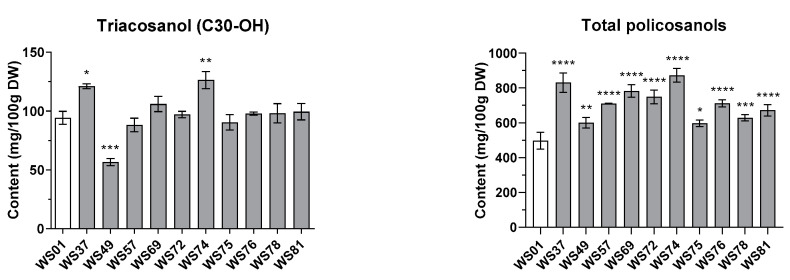
Policosanol contents (mg/100 g dry weight (DW)) in the sprouts of the original wheat cultivar and its 10 selected mutant lines. * (*p* < 0.05), ** (*p* < 0.005), *** (*p* < 0.001), and **** (*p* < 0.0001) indicate statistical significance.

To develop new varieties of *T. aestivum* for sprouts containing high contents of functional ingredients, the top 10 mutant lines with a >20% higher total PC content than that of the original cultivar were selected. The GC-MS chromatograms for each sample are shown in Figure S2. The accumulation patterns of individual components differed owing to different radiation breeding methods (Figure 2). The octacosanol (C28-OH) content increased in the top 10 mutant lines but showed a varied trend in the other 75 mutant lines. Moreover, the triacontanol (C30-OH) content increased in only 7 out of the top 10 mutant lines, except for three lines (WS49, WS57, and WS75) but decreased in the other 75 mutant lines. The tricosanol (C23-OH) content increased in all the mutant lines except for WS37 and WS56. The contents of the other four compounds showed varied increasing and decreasing trends.

Among the 10 selected mutant lines, the highest total PC content was observed in WS74 (873.24 mg/100 g). The octacosanol (C28-OH; 686.38 mg/100 g), tricosanol (C23-OH; 13.61 mg/100 g), and docosanol (C22-OH; 9.94 mg/100 g) contents in WS74 were more than twice as high as those in WS01, and the content of the remaining four components slightly increased (<1.3 times) or decreased. WS37 ranked second in the total PC content, and its octacosanol (C28-OH; 636.02 mg/100 g), hexacosanol (C26-OH; 58.12 mg/100 g), and triacontanol (C30-OH; 121.20 mg/100 g) contents increased by ~1.8, 1.7, and 1.3 times, respectively, compared to those in WS01; however, the contents of the other compounds were similar to those in WS01 or decreased. WS76 had the highest contents of docosanol (C22-OH; 14.10 mg/100 g) and tetracosanol (C24-OH; 7.46 mg/100 g), which were more than three times higher than those in WS01. Furthermore, WS53 contained the highest contents of hexacosanol (C26-OH; 70.85 mg/100 g) and heptacosanol (C27-OH; 16.25 mg/100 g), which were twice as high as those in WS01. The highest tricosanol (C23-OH) content (17.07 mg/100 g) was observed in WS49. These results suggest that γ-irradiation breeding influences genetic mutation for the increased accumulation of beneficial metabolites, as previously reported for wheat and perilla [5,40].

Compared to the commercially available cultivars cv. Geumkang (WS87) and cv. Cheongwoo (WS91), the original cultivar (Woori-mil × D-7; WS01), had a higher total PC content. However, compared to those in WS01 and WS87, WS91 showed 60–70% lower octacosanol (C28-OH) and triacontanol (C30-OH) contents, which are major components, and slightly higher tricosanol (C23-OH), tetracosanol (C24-OH), and heptacosanol (C27-OH) contents. The three mutant lines (WS88–WS90) derived from cv. Geumkang (WS87) had a slightly lower total PC content than that in WS87. WS88 and WS90 showed a similar PC content distribution to that in WS87. However, WS89 had 1.85 times the tricosanol (C23-OH) present in WS87, but the contents of the other components decreased compared to those in WS87.

### 2.3. Effects of Different LED Conditions on the Policosanol Content in the Sprouts of Wheat Mutant Lines

**Figure 3:**
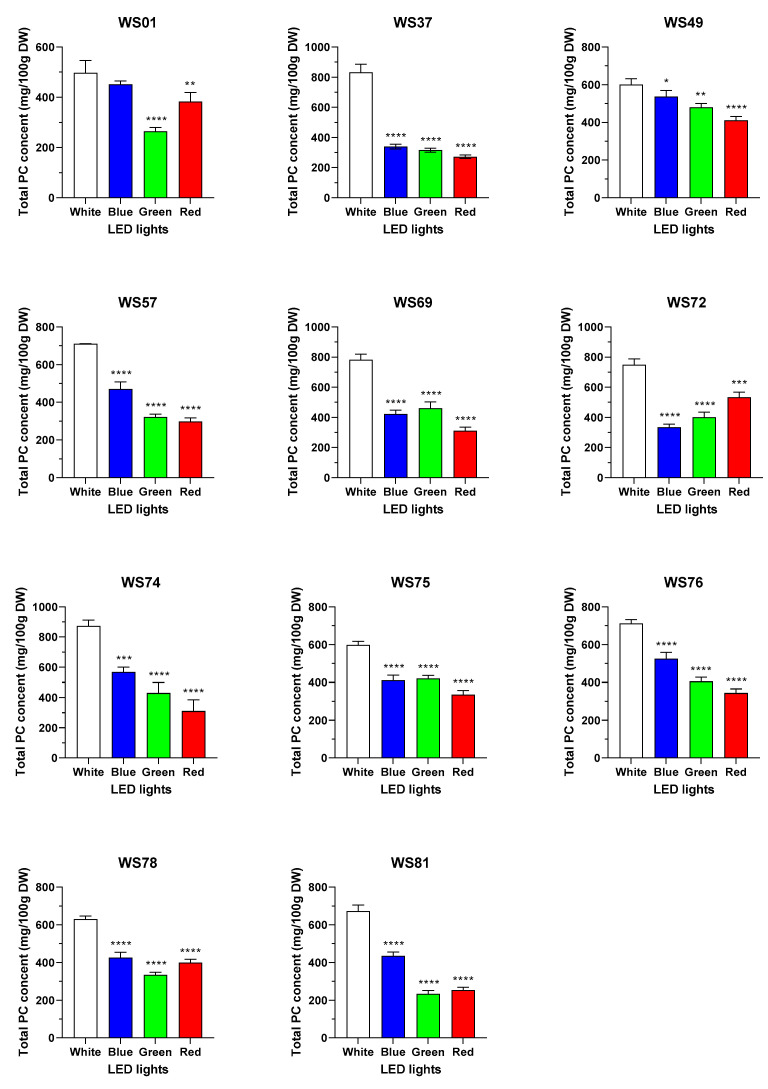
Total policosanol content (mg/100 g dry weight (DW)) in the sprouts of the original cultivar and its 10 selected mutant lines under different LED light conditions. * (*p* < 0.05), ** (*p* < 0.005), *** (*p* < 0.001), and **** (*p* < 0.0001) indicate statistical significance.

To evaluate the effects of different LED conditions on PC accumulation in wheat sprouts, we quantified the PC content in the top 10 wheat mutant lines and the original variety, which were grown in a well-controlled growth chamber under white (6000 K), blue (440 nm), green (520 nm), and red (660 nm) LED light irradiation for 7 d, using GC-MS (Table 3, Table 4 and Table 5). The GC-MS chromatograms for the PCs and each sample are shown in Figures S3–S6. The total and individual PC contents significantly decreased in the wheat sprout samples irradiated with blue, green, and red LED lights compared to the PC contents in the samples irradiated with a white LED light; in particular, the total PC contents in the sample irradiated with blue, green, and red light decreased by 35.8, 46.9, and 49.7%, respectively (Figure 3).

The total PC content in the original variety WS01 decreased more under green-light irradiation than under blue- or red-light irradiation. WS78 and WS81 exhibited similar patterns to WS01, whereas the total PC content in WS37, WS49, WS57, WS74, and WS76 decreased slightly as the wavelength of the colored LED lights increased in the order of blue (440 nm) < green (520 nm) < red (660 nm). In contrast, the total PC content in WS72 showed a slight decreasing trend as the wavelength of the colored LED lights decreased from red to green to blue. The total PC content in WS69 and WS75 decreased slightly under green LED light compared to that under blue and red LED lights. Hence, the individual and total PC content in wheat sprout samples varied without a specific trend under different LED light conditions, and white LED light was the best condition for PC accumulation. The inconsistent or negative changes in the PC content under LED cultivation observed in this study were similar to those reported in previous studies on barley and wheat sprouts [7]. Irregular trends of hexacosanol (C26-OH) and octacosanol (C28-OH) accumulation were observed in barley and wheat sprout samples, respectively, cultivated under fluorescent and white, blue, and red LED irradiation on the 7th day of growth. This suggests that light irradiation is not the only factor that influences PC biosynthesis.

## 3. Discussions

Wheat (*T. aestivum*) is one of the most important energy sources; however, its sprout has received considerable attention due to its nutritional and functional aspects. PCs are a mixture of primary alcohols of a very long-chain fatty acid consisting of eicosanol (C20-OH), heneicosanol (C21-OH), docosanol (C22-OH), tricosanol (C23-OH), tetracosanol (C24-OH), hexacosanol (C26-OH), heptacosanol (C27-OH), octacosanol (C28-OH), and triacontanol (C30-OH). They are found in cuticular waxes in sprouts of cereal crops and waxes of insects. Among them, octacosanol (C28-OH) was the most abundant PC in wheat sprouts [6], whereas hexacosanol (C26-OH) is the most abundant PC in other cereals, barley, and oats [25,26]. In the PC profiles of the sprouts of 17 Korean wheat cultivars, their PC contents on the 6th day of growth was the highest, and the average content of the total PCs in the six days of 17 cultivars was 738 mg/100 g [6]. In this study, the average content of the total PCs in the 6 days of the top ten improved mutant lines was 716 mg/100 g, which is comparable to the results of the previously reported value. Various other natural sources have also been identified as potential sources of PCs. Sugar cane peel mainly contained octacosanol (C28-OH; 81%), and its leaves had a high content ratio of not only octacosanol (C28-OH; 46%) but also tetracosanol (C24-OH; 16%), triacontanol (C30-OH; 15%), and hexacosanol (C26-OH; 12%) [41]. The main PC component of beeswax was triacontanol (C30-OH), accounting for approximately 45% [41]. For adley (*Coix lacryma-jobi),* the main components were docosanol (C22-OH), tetracosanol (C24-OH), hexacosanol (C26-OH), and octacosanol (C28-OH) [42]. Corn kernels contained dotriacontanol (C32-OH), followed by triacontanol (C30-OH) and tetracosanol (C24-OH) [43]. The most representative compounds in hemp (*Cannabis sativa*) were tetracosanol (C24-OH), hexacosanol (C26-OH), and dotriacontanol (C32-OH) [13]. The main PC component found in peanut kernels (*Arachis hypogaea*) was docosanol (C22-OH) [44]. For Korean winter spinach (*Spinacia oleracea*), the major components were tetracosanol (C24-OH) and hexacosanol (C26-OH) [45]. In this study, the total PC content of the improved mutant line W74 sample was 873 mg/100 g, which was higher than that of the other sources of PC production cited in the literature, such as sugar cane, in which the total PC level is 181–270 mg/100 g; beeswax, 5–12 mg/100 g; adley, 89–246 mg/100 g; corn kernel, 16–20 mg/100 g; hemp, 397–608 mg/100 g; peanut, 38–54 mg/100 g; and winter spinach, 53–59 mg/100 g [13,40–45]. Therefore, the data on the quantification of PCs in the sprouts of improved wheat mutant lines suggest a beneficial value of wheat mutant lines as a natural source rich in PCs.

Previous studies on the biosynthesis of long-chain primary alcohols found in wheat sprout cuticle wax have identified and revealed new functions of fatty acyl coenzyme A reductase (FAR) [38]. TaFAR5, which encodes FAR responsible for forming primary alcohol in wheat leaf blades, was identified. Heterologous expression in yeast (*Saccharomyces cerevisiae*) led to the production of C22-OH [38]. Additionally, when TaFAR6, TaFAR7, and TaFAR8, identified in the wheat leaf epidermis, were heterologously expressed in yeast (*S. cerevisiae*), C24-OH and C26-OH were produced [46]. However, the transgenic expression of these three TaFARs in tomato (*Solanum lycopersicum*) and rice (*O. sativa*) increased the accumulation of C24-OH–C30-OH [46]. Five wax biosynthetic genes, Ae.tFAR1, Ae.tFAR2, Ae.tFAR3, Ae.tFAR4, and Ae.tFAR6, identified in the genome of the *Aegilops tauschii* leaf surface, a heterologous expression of five Ae.tFARs in yeast (*S. cerevisiae*) which resulted in Ae.tFAR1, Ae.tFAR2, Ae.tFAR3, Ae.tFAR4, and Ae.tFAR6, produced C16-OH, C18-OH, C26-OH, C24-OH, and C28-OH, respectively [39]. Three Brachypodium FAR genes, BdFAR1, BdFAR2, and BdFAR3, were isolated from the leaf wax of *Brachypodium distachyon*, and the transgenic BdFAR2 and BdFAR3 lines of Brachypodium showed significantly increased amounts of C26-OH and C28-OH compared to the wild species [47]. From available barley (*Hordeum vulgare*) genome information, HvFAR2, HvFAR3, HvFAR4, HvFAR5, and HvFAR6 were identified, and the HvFAR3 expression increased the accumulation of C26-OH in barley sprouts [7]. Therefore, TaFAR6, TaFAR7, TaFAR8, Ae.tFAR6, BdFAR2, and BdFAR3 are possibly involved in the biosynthesis of octacosanol (C28-OH), which is a significant component in wheat sprouts.

The content of octacosanol (C28-OH), which was the major constituent of wheat sprouts in this study, increased in the top 10 mutant lines (W74, WS37, WS69, WS72, WS76, WS57, WS81, WS78, WS49, and WS75) along with an increased total PC content, compared to the original variety. Octacosanol (C28-OH) induces a dose-dependent activation of AMPK phosphorylation in human hepatoma HepG2 cells, suggesting it is a lipid/cholesterol-lowering agent [6,48]. In addition, new molecular mechanisms of octacosanol (C28-OH) have recently been discovered, including insulin-resistance management by the regulation of the gut microbiota and inflammatory signaling pathway [49], lipid-decreasing effects through a modulation of the lipid metabolism-related signaling pathway [50], protective effects on the integrity of the gut barrier through a modulation of the intestinal flora and its metabolism [51], and anti-fatigue effects in an exercise-induced fatigue model [52]. Therefore, mutant lines exhibiting a high octacosanol (C28-OH) accumulation could have potential applications as functional foods and promote new cultivars’ development.

Red/far-red light-sensing phytochromes and blue light-sensing cryptochromes play important roles in regulating light-mediated physiological responses through regulated transcriptional networks [53]. Phytochromes are photoreceptors that display a photoreversible conformational change between two spectrally distinct forms: red light absorbing Pr and far-red light-absorbing Pfr. Red light converts Pr to biologically active Pfr, inducing reactions such as seed germination and desulfurization, while far-infrared light converts Pfr back to Pr, physically inactivating it [27,53]. Cryptochromes with B-light photoreceptor functions have two members, cry1 and cry2. Both cryptochromes undergo B light-dependent phosphorylation, and homodimerization is required for these cryptochromes’ phosphorylation and physiological activity [27,53]. In particular, it was revealed that cry2 plays a role in forming homodimers in response to blue light [54]. Therefore, applying light resources is promising to improve crop biomass and quality. The light environment is a factor that has a significant impact on the production of secondary metabolites in plants [55].

Additionally, growing plants under different wavelengths of light exposure results in physiological changes [56]. For example, it has been reported that the type and intensity of the wavelength can affect the enhancement of carotenoid pigment and glucosinolate concentrations [57,58]. Conversely, a GC-MS analysis of the PC content in sprouts of barley (*H. vulgare*) and wheat (*T. aestivum*) subjected to differential LED light conditions showed that the hexacosanol (C26-OH) content in barley and the octacosanol (C28-OH) content in wheat were not consistent with light qualities [7]. In this study, we attempted to enhance the policosanol content of wheat sprouts using different wavelengths of light. The top 10 mutant lines with the highest increase in total PC content were cultivated under blue, green, and red LED light irradiation to investigate the effect of LED irradiation on the production of PCs. However, the total PC content decreased or showed irregular trends, suggesting that the white LED light irradiation was the most effective condition for increasing PC production. In addition, it indicated that LED responses may be crop- or secondary metabolite-dependent and that a colored LED light is not a factor in amplifying PC biosynthesis in wheat sprouts.

In conclusion, the differences in the individual and total PC contents of the wheat sprout samples, including the original variety (WS01), commercially available varieties cv. Geumgang (WS87) and cv. Cheongwoo (WS91), and 85 mutant lines (WS02–WS86 and WS88–WS90) were evaluated for the first time in the literature. Compared to WS01, 18 mutant lines exhibited a higher total PC content with an increased octacosanol (C28-OH) content, suggesting that these mutant lines can serve as a functional source of PCs. Moreover, the top 10 mutant lines with the highest range of a total PC were subjected to differential LED light conditions (blue, green, and red). However, their individual and total PC contents were reduced compared to those with the white LED light irradiation, indicating that the correlation between colored LED lights and PC biosynthesis in wheat sprouts is negative. Our study reveals the beneficial effect of radiation breeding on increasing the metabolite accumulation in wheat sprouts. However, further studies on the genetic factors controlling radiation-induced PC biosynthesis in wheat sprouts are required.

## 4. Materials and Methods

### 4.1. Plant Materials

Wheat mutant lines were developed by radiation breeding of the original cultivar of colored wheat using seeds treated with 200 Gy of gamma (^60^Co) irradiation. The original cultivar of colored wheat was developed by a cross-breeding of Woori-mil (Korea RDA accession no. IT172221) and D-7 (a wheat line developed by Korea University) [59]. The mutant lines selected according to the phenotypic variants stably inherited their phenotype for over 4 years and were bred by Drs. Jin-Beak Kim and Min-Jeong Hong (Korea Atomic Energy Research Institute). Voucher specimens were deposited at the Radiation Breeding Research Center, the Advanced Radiation Technology Institute, and the Korea Atomic Energy Research Institute. In this study, seeds of the selected mutants were sown in 50-cell seeding plug trays with soil and water and were germinated and grown in a well-controlled growth chamber (DS-50TPLH-3Light; Dasol Science, Hwaseong-si, Gyeonggi-dom, Republic of Korea) at 22 °C with 60% relative humidity. The growth chambers were irradiated with white (6000 K), blue (440 nm), green (520 nm), and red (660 nm) lights with a photoperiod of 16 h of light and 8 h of darkness. Sprouts of the wheat mutant lines were collected 7 d after sowing. Each sample was freeze-dried, chopped, and stored at −20 °C in polyethylene plastic bags until further analysis.

### 4.2. Sample Preparation

For the quantitative analysis of the PCs, sprouts (0.1 g) of wheat samples cultivated in a growth chamber under a white LED light (6000 K) were extracted into 10 mL of hexane with shaking at 24 °C for 24 h. Each hexane extract was filtered and evaporated under a vacuum. Chloroform (0.5 mL) and 250 μL of *N*-methyl-*N*-(trimethylsilyl) trifluoroacetamide (MSTFA; Sigma-Aldrich) were added to each hexane extract for the silylation of the PCs [21]. The solutions were then incubated in a water bath at 50 °C for 15 min. The PCs (>97% purity) eicosanol (C20), heneicosanol (C21), docosanol (C22), tricosanol (C23), tetracosanol (C24), hexacosanol (C26), heptacosanol (C27), octacosanol (C28), and triacosanol (C30) were obtained from Sigma-Aldrich (St. Louis, MO, USA). These compounds were also derivatized with MSTFA into PC-TMS derivatives for GC analysis. Calibration curves were prepared using a series of standard solutions at four different concentrations (2, 5, 10, and 20 μg/mL or 5, 10, 20, and 50 μg/mL). The relationships between the peak areas (*y*) and concentrations (*x*, μg/mL) of the PC standards were calculated using second-degree polynomial regression equations (*y* = *ax* + *b*; *a*: slope; *b*: intercept), and their correlation coefficients are shown below: eicosanol (C20; *y* = −560.83*x*^2^ + 209215*x* − 222658, *R*² = 0.9995); heneicosanol (C21; *y* = −364.13*x*^2^ + 176656*x* − 131770, *R*² = 0.9998); docosanol (C22; *y* = 2179.3*x*^2^ + 115046*x* + 28299, *R*² = 0.9999); tricosanol (C23; *y* = 1574*x*^2^ + 89951*x* + 17643, *R*² = 1); tetracosanol (C24; *y* = 169.85*x*^2^ + 110474*x* − 103988, *R*² = 1); hexacosanol (C26; *y* = 783.01*x*^2^ + 66215*x* − 93374, *R*² = 0.9997); heptacosanol (C27; *y* = 842.49*x*^2^ + 53175*x* − 149882, *R*² = 1); octacosanol (C28; *y* = 338.49*x*^2^ + 49097*x* − 198688, *R*² = 0.9981); and triacosanol (C30; *y* = 311.7*x*^2^ + 9044.2*x* + 2673.2, *R*² = 1).

Wheat sprouts cultivated in a growth chamber irradiated with blue (440 nm), green (520 nm), and red (660 nm) LED lights were extracted and silylated using the procedure described above. Calibration curves were prepared using a series of standard solutions at four different concentrations. The regression equations of the calibration curves of the PCs and the coefficients were calculated as follows: eicosanol (C20; *y* = 75727*x* − 17152, *R*² = 0.9997); heneicosanol (C21; *y* = 61098*x* +35161, *R*² = 0.9994); docosanol (C22; *y* = 63960*x* − 3863.4, *R*² = 0.9992); tricosanol (C23; *y* = 60937*x* − 9030.1, *R*² = 0.9999); tetracosanol (C24; *y* = 46824*x* + 1555.3, *R*² = 0.9983); hexacosanol (C26; *y* = 39868*x* − 24041, *R*² = 0.9971); heptacosanol (C27; *y* = 49217*x* + 4888.4, *R*² = 0.9997); octacosanol (C28; *y* = 39362*x* + 85896, *R*² = 0.9956); and triacosanol (C30; *y* = 37712*x* + 12246, *R*² = 0.9968).

### 4.3. GC-MS Analysis

The GC-MS analysis of wheat sprout samples cultivated in a growth chamber under a white LED light (6000 K) was performed on a GCMS-QP2010 Ultra (Shimadzu, Kyoto, Japan). The GC-MS analysis of wheat sprout samples cultivated in a growth chamber with blue, green, and red LED lights was performed using a GCMS-QP2020 NX (Shimadzu). An HP-5 MS capillary GC column (30 m length × 0.25 mm i.d. × 0.25 μm film thickness, Agilent Technologies Co., Santa Clara, CA, USA) was used with 99.99% high-purity helium at a flow rate of 1.2 mL/min. The sample (1 μL) was injected in split mode (1:5 ratio). The oven temperature was initially set to 230 °C, was increased to 260 °C at a heating rate of 25 °C/min, and was maintained at this temperature for 10 min. The transfer line temperature was set to 280 °C. The MS data were acquired in the electron ionization (EI) mode with an ionization voltage of 70 eV, an ion source temperature of 230 °C, and a scan range of *m*/*z* 50–500. The MS data were collected using a mass spectra database (National Institute of Standards and Technology, MassSpectra Libraries, Gaithersburg, MD, USA). The PCs were identified by comparing the retention time and fragmented mass values of the peaks with those of the PC standards.

### 4.4. Statistical Analysis

All experiments were conducted in triplicate, and the mean values were reported. One-way analysis of variance was performed using GraphPad Prism 9 software (GraphPad Software, La Jolla, CA, USA) to assess significant differences (* *p* < 0.05, ** *p* < 0.005, *** *p* < 0.001, and **** *p* < 0.0001). The data set of the quantification of the PCs obtained for the 91 wheat sprout samples was normalized and subjected to a hierarchical clustering analysis (HCA) with a heatmap using “pheatmap” in R software (version 4.0.2) [60].

## Figures and Tables

**Table 1:** GC-MS retention times and mass spectral data of the policosanols.

*t*_R_ (min)	PC-TMS Derivatives ^1^ Mass (*m*/*z*)	Molecular Weight (*m*/*z*)	Molecular Formula	Identification
3.57	355.3 [M–15]^+^	370.3 [M]^+^	C_20_H_42_O	Eicosanol
4.20	369.4 [M–15]^+^	384.4 [M]^+^	C_21_H_44_O	Heneicosanol
5.02	383.4 [M–15]^+^	398.4 [M]^+^	C_22_H_46_O	Docosanol
5.96	397.4 [M–15]^+^	412.4 [M]^+^	C_23_H_48_O	Tricosanol
7.38	411.4 [M–15]^+^	426.4 [M]^+^	C_24_H_50_O	Tetracosanol
11.30	439.5 [M–15]^+^	454.5 [M]^+^	C_26_H_54_O	Hexacosanol
12.83	453.5 [M–15]^+^	468.5 [M]^+^	C_27_H_56_O	Heptacosanol
13.95	467.5 [M–15]^+^	482.5 [M]^+^	C_28_H_58_O	Octacosanol
16.43	495.5 [M–15]^+^	510.5 [M]^+^	C_30_H_62_O	Triacontanol

^1^ Trimethylsilane (TMS) derivatization of policosanols (PCs).

**Table 2:** Individual and total policosanol contents in the sprouts of wheat mutant lines.

Cultivars	Mutant Lines	Policosanol Content (mg/100 g Dry Weight) ^a,b^							
		C22-OH ^c^	C23-OH ^c^	C24-OH ^c^	C26-OH ^c^	C27-OH ^c^	C28-OH ^c^	C30-OH ^c^	Total PC
Woori-mil × D-7 (original; WS01)		4.72 ± 3.13	5.94 ± 0.64	1.94 ± 0.33	35.02 ± 1.99	6.98 ± 1.03	348.57 ± 34.81	94.34 ± 9.37	497.50 ± 51.30
	WS02	4.19 ± 3.46	11.05 ± 3.94	4.31 ± 3.03	42.49 ± 10.64	10.75 ± 5.22	247.91 ± 32.35	49.94 ± 25.61	370.63 ± 84.25
	WS03	5.99 ± 2.33	13.39 ± 3.08	6.89 ± 2.65	46.89 ± 9.27	11.99 ± 5.85	212.92 ± 14.99	63.09 ± 15.34	361.17 ± 53.50
	WS04	2.81 ± 0.70	7.24 ± 0.94	1.88 ± 0.40	29.97 ± 6.20	5.65 ± 1.00	268.99 ± 17.92	60.02 ± 12.46	376.56 ± 39.62
	WS05	6.02 ± 3.48	6.75 ± 0.97	3.07 ± 1.87	41.35 ± 8.54	16.98 ± 18.85	321.24 ± 34.29	77.08 ± 17.67	472.48 ± 85.67
	WS06	3.49 ± 2.63	7.911.93	1.921.89	22.93 ± 2.87	10.15 ± 0.62	223.92 ± 19.73	69.33 ± 9.55	339.65 ± 39.22
	WS07	3.71 ± 0.91	9.10 ± 0.82	5.65 ± 0.93	31.49 ± 4.23	10.17 ± 0.89	233.01 ± 26.73	67.32 ± 10.39	360.46 ± 44.90
	WS08	4.33 ± 0.37	10.29 ± 1.36	7.10 ± 0.98	43.15 ± 5.09	10.60 ± 1.29	227.97 ± 12.36	58.02 ± 9.66	361.45 ± 31.13
	WS09	3.68 ± 1.92	11.21 ± 1.38	4.08 ± 2.18	29.01 ± 3.20	5.80 ± 1.60	249.19 ± 13.68	76.01 ± 7.44	378.98 ± 31.39
	WS10	5.03 ± 1.92	8.44 ± 0.59	2.32 ± 1.01	29.02 ± 2.27	7.10 ± 4.29	228.27 ± 14.28	65.21 ± 7.02	345.39 ± 31.39
	WS11	8.34 ± 2.69	12.75 ± 1.37	6.69 ± 1.86	32.47 ± 1.96	6.47 ± 1.54	283.89 ± 25.47	86.73 ± 6.81	437.34 ± 41.70
	WS12	3.26 ± 0.60	8.69 ± 0.81	2.10 ± 0.44	23.41 ± 1.52	11.09 ± 4.04	214.56 ± 18.32	68.22 ± 5.39	331.32 ± 31.11
	WS13	4.39 ± 1.10	9.51 ± 0.63	9.73 ± 1.06	52.22 ± 3.74	12.52 ± 2.50	214.25 ± 11.12	62.62 ± 2.42	365.25 ± 22.58
	WS14	4.10 ± 2.10	9.24 ± 0.14	5.93 ± 0.41	35.02 ± 2.10	6.41 ± 1.23	234.20 ± 24.78	73.44 ± 6.40	368.34 ± 37.15
	WS15	6.18 ± 0.85	8.18 ± 0.75	8.82 ± 1.25	39.30 ± 2.92	9.01 ± 3.83	182.72 ± 31.71	62.24 ± 9.76	316.46 ± 51.07
	WS16	1.06 ± 0.24	10.76 ± 1.07	3.48 ± 0.46	42.65 ± 2.96	9.38 ± 0.64	248.19 ± 22.15	68.76 ± 4.29	384.27 ± 31.81
	WS17	2.92 ± 1.32	8.65 ± 0.57	3.90 ± 2.07	30.11 ± 1.52	5.30 ± 1.64	240.05 ± 28.98	73.86 ± 3.70	364.79 ± 39.80
	WS18	6.46 ± 2.43	12.00 ± 1.25	4.25 ± 2.09	27.38 ± 1.60	6.67 ± 0.49	264.94 ± 24.32	78.53 ± 6.25	400.23 ± 38.45
	WS19	2.13 ± 0.26	6.75 ± 0.48	3.15 ± 0.14	43.46 ± 2.66	7.89 ± 1.19	270.22 ± 27.60	74.98 ± 7.16	408.57 ± 39.49
	WS20	3.43 ± 0.24	9.56 ± 0.83	2.06 ± 0.09	38.58 ± 1.54	6.50 ± 0.95	222.01 ± 20.38	37.65 ± 2.16	319.79 ± 26.20
	WS21	3.16 ± 1.68	11.73 ± 0.98	4.84 ± 1.98	32.11 ± 0.99	5.68 ± 0.82	248.80 ± 23.24	74.36 ± 3.87	380.67 ± 33.56
	WS22	3.86 ± 2.18	11.02 ± 1.02	1.53 ± 0.25	26.90 ± 1.05	5.98 ± 3.09	241.09 ± 29.59	73.14 ± 4.35	363.51 ± 41.52
	WS23	1.31 ± 1.28	6.31 ± 5.47	0.23 ± 0.20	19.11 ± 16.56	3.33 ± 5.14	205.24 ± 178.40	53.64 ± 46.83	289.17 ± 253.88
	WS24	2.67 ± 0.61	8.83 ± 0.41	3.04 ± 0.09	29.91 ± 1.03	4.81 ± 0.89	242.92 ± 27.91	69.96 ± 4.80	362.14 ± 35.74
	WS25	4.18 ± 0.71	10.34 ± 0.72	3.58 ± 0.23	33.03 ± 1.11	7.06 ± 0.46	224.44 ± 9.35	56.60 ± 3.17	339.23 ± 15.75
	WS26	1.95 ± 0.35	8.31 ± 0.68	3.03 ± 0.13	37.80 ± 1.95	11.19 ± 4.43	183.26 ± 17.87	56.61 ± 4.45	302.14 ± 29.85
	WS27	2.37 ± 2.42	10.11 ± 0.80	0.94 ± 0.49	39.59 ± 1.31	10.51 ± 4.38	312.25 ± 13.62	60.60 ± 10.97	436.37 ± 34.00
	WS28	4.80 ± 1.39	8.47 ± 0.79	1.52 ± 0.21	28.63 ± 1.43	6.75 ± 0.67	283.63 ± 28.49	60.79 ± 5.78	394.59 ± 38.75
	WS29	5.33 ± 0.11	14.71 ± 1.25	5.36 ± 1.57	43.33 ± 1.30	5.62 ± 1.46	333.42 ± 30.30	80.04 ± 6.35	487.81 ± 42.35
	WS30	2.37 ± 1.13	9.09 ± 0.81	4.51 ± 1.28	44.15 ± 2.22	10.79 ± 1.04	211.24 ± 10.40	65.61 ± 4.95	347.76 ± 21.82
	WS31	4.35 ± 0.68	12.07 ± 0.36	4.64 ± 1.61	60.96 ± 2.13	10.82 ± 3.30	390.60 ± 36.83	98.94 ± 7.69	582.37 ± 52.60
	WS32	3.18 ± 0.01	7.43 ± 0.36	3.10 ± 0.20	37.94 ± 2.06	9.79 ± 2.11	202.02 ± 14.89	63.54 ± 1.07	326.99 ± 20.70
	WS33	5.16 ± 0.91	7.67 ± 0.65	5.47 ± 1.58	45.34 ± 2.74	7.64 ± 0.88	213.42 ± 12.63	69.59 ± 3.46	354.29 ± 22.86
	WS34	1.36 ± 0.59	9.97 ± 0.60	2.64 ± 0.83	26.81 ± 0.82	3.76 ± 0.48	224.64 ± 19.59	59.22 ± 3.72	328.41 ± 26.63
	WS35	3.39 ± 1.93	7.21 ± 0.52	3.42 ± 0.20	42.70 ± 1.95	6.03 ± 1.17	223.93 ± 26.32	67.28 ± 2.58	353.96 ± 34.67
	WS36	2.11 ± 1.11	8.62 ± 0.41	0.90 ± 0.11	23.35 ± 0.50	9.33 ± 3.12	209.56 ± 22.28	62.47 ± 2.26	316.33 ± 29.79
	WS37	4.37 ± 2.50	5.88 ± 0.34	0.53 ± 0.35	58.12 ± 4.12	4.88 ± 2.91	636.02 ± 43.59	121.20 ± 3.70	830.99 ± 57.51
	WS38	1.84 ± 1.61	6.78 ± 6.00	6.80 ± 5.93	29.65 ± 25.83	8.52 ± 7.62	130.34 ± 114.73	40.44 ± 35.37	224.38 ± 197.10
	WS39	3.42 ± 0.05	8.67 ± 0.33	2.24 ± 0.21	29.21 ± 0.89	12.15 ± 5.67	239.92 ± 19.22	60.83 ± 5.32	356.43 ± 31.68
	WS40	2.93 ± 1.69	13.44 ± 0.63	2.49 ± 3.32	31.93 ± 1.35	7.55 ± 3.46	372.22 ± 24.89	75.52 ± 11.07	506.08 ± 46.41
	WS41	3.65 ± 1.33	13.39 ± 0.49	5.46 ± 1.80	45.97 ± 1.44	11.73 ± 5.76	290.41 ± 23.28	73.77 ± 1.49	444.37 ± 35.60
	WS42	3.18 ± 0.05	10.78 ± 0.23	10.59 ± 0.32	49.75 ± 0.13	15.39 ± 1.00	234.83 ± 26.43	59.00 ± 2.41	383.53 ± 30.57
	WS43	3.77 ± 2.35	12.58 ± 0.28	6.23 ± 1.76	33.30 ± 0.86	9.53 ± 3.43	338.65 ± 25.86	67.91 ± 7.65	471.95 ± 42.19
	WS44	2.24 ± 0.14	12.46 ± 0.38	4.26 ± 2.33	29.30 ± 0.40	5.82 ± 1.05	324.63 ± 24.16	64.26 ± 5.46	442.97 ± 33.94
	WS45	3.00 ± 0.14	11.54 ± 0.34	3.29 ± 2.13	25.95 ± 0.31	5.54 ± 1.44	317.86 ± 12.59	78.58 ± 2.36	445.77 ± 19.32
	WS46	5.76 ± 0.10	11.46 ± 0.27	7.10 ± 0.57	63.67 ± 0.99	9.75 ± 3.40	395.49 ± 20.94	65.42 ± 8.83	558.65 ± 35.10
	WS47	3.04 ± 0.07	9.28 ± 0.19	1.49 ± 0.18	26.71 ± 0.94	10.18 ± 4.17	291.95 ± 19.80	70.68 ± 0.47	413.34 ± 25.84
	WS48	4.33 ± 0.13	11.87 ± 0.33	2.35 ± 0.23	31.40 ± 0.56	10.54 ± 5.92	417.83 ± 30.78	67.92 ± 11.63	546.24 ± 49.57
	WS49	4.68 ± 0.09	17.07 ± 0.41	4.03 ± 0.36	65.12 ± 1.88	7.87 ± 1.21	445.23 ± 33.53	56.78 ± 5.16	600.78 ± 42.64
	WS50	2.40 ± 1.09	10.00 ± 0.04	1.30 ± 0.15	26.84 ± 0.67	5.98 ± 1.36	279.45 ± 26.89	71.29 ± 4.22	397.24 ± 34.42
	WS51	5.24 ± 0.10	10.86 ± 0.85	4.17 ± 0.54	57.39 ± 1.88	10.36 ± 3.37	403.45 ± 26.90	68.34 ± 3.02	559.80 ± 36.67
	WS52	2.04 ± 0.16	7.75 ± 0.34	0.65 ± 0.08	23.36 ± 0.69	8.37 ± 2.73	324.61 ± 22.25	75.97 ± 3.06	442.75 ± 29.30
	WS53	4.20 ± 0.11	12.31 ± 0.17	5.52 ± 0.30	54.09 ± 0.36	11.51 ± 0.82	340.89 ± 24.48	69.13 ± 1.76	497.64 ± 27.99
	WS54	4.30 ± 0.13	9.96 ± 0.28	8.57 ± 1.80	60.26 ± 0.36	18.91 ± 0.42	271.48 ± 42.23	71.55 ± 0.22	445.03 ± 45.45
	WS55	4.03 ± 0.13	9.35 ± 0.18	1.80 ± 0.17	25.73 ± 1.06	5.73 ± 0.45	357.13 ± 27.72	64.08 ± 0.97	467.84 ± 30.68
	WS56	2.25 ± 0.24	5.58 ± 0.38	1.65 ± 0.42	26.90 ± 0.36	13.11 ± 2.32	278.15 ± 20.14	72.06 ± 2.81	399.69 ± 26.67
	WS57	6.32 ± 4.38	7.66 ± 1.00	5.27 ± 3.56	70.85 ± 0.91	16.25 ± 1.49	515.98 ± 13.87	88.27 ± 9.94	710.60 ± 35.15
	WS58	2.37 ± 0.09	14.26 ± 0.65	1.66 ± 0.38	31.34 ± 0.56	7.80 ± 4.51	328.16 ± 24.62	71.32 ± 2.71	456.90 ± 33.52
	WS59	8.77 ± 0.32	8.20 ± 0.32	2.73 ± 0.23	18.95 ± 0.54	6.46 ± 0.78	296.17 ± 22.91	61.64 ± 2.26	402.93 ± 27.36
	WS60	3.17 ± 0.01	7.15 ± 0.19	4.08 ± 0.17	32.73 ± 0.70	12.12 ± 5.33	225.04 ± 14.92	63.22 ± 3.97	347.50 ± 25.28
	WS61	6.28 ± 0.22	12.93 ± 0.05	4.99 ± 0.21	44.79 ± 0.56	12.08 ± 3.25	287.80 ± 40.92	73.70 ± 0.79	442.58 ± 46.00
	WS62	1.86 ± 0.04	12.91 ± 0.18	2.46 ± 0.80	28.47 ± 0.51	7.91 ± 3.96	334.24 ± 26.90	79.09 ± 1.18	466.95 ± 33.58
	WS63	2.80 ± 0.10	10.56 ± 0.12	0.00 ± 0.00	15.32 ± 1.84	4.35 ± 4.45	456.98 ± 20.46	66.07 ± 5.03	556.09 ± 32.00
	WS64	9.68 ± 1.61	14.38 ± 0.37	3.02 ± 0.17	32.92 ± 0.23	5.74 ± 1.70	350.95 ± 25.99	70.70 ± 9.45	487.40 ± 39.52
	WS65	5.61 ± 0.87	13.36 ± 0.30	10.37 ± 1.11	51.54 ± 0.42	15.02 ± 0.95	269.75 ± 27.57	61.73 ± 8.60	427.39 ± 39.80
	WS66	9.96 ± 0.16	26.47 ± 0.28	3.84 ± 0.39	33.61 ± 0.49	6.58 ± 1.92	377.49 ± 26.68	50.85 ± 2.96	508.80 ± 32.90
	WS67	3.98 ± 2.84	14.67 ± 1.23	6.48 ± 2.72	25.89 ± 1.34	7.93 ± 3.84	284.14 ± 20.94	75.83 ± 5.57	418.92 ± 38.49
	WS68	4.46 ± 0.11	11.87 ± 0.12	8.78 ± 2.54	49.24 ± 1.15	12.87 ± 3.56	273.23 ± 15.29	67.80 ± 2.35	428.24 ± 25.13
	WS69	9.15 ± 0.51	15.91 ± 0.52	3.11 ± 0.12	24.93 ± 7.79	5.73 ± 2.45	617.49 ± 33.86	106.11 ± 11.28	782.42 ± 56.53
	WS70	2.94 ± 0.11	9.81 ± 0.27	1.14 ± 0.24	29.41 ± 0.58	12.48 ± 4.07	394.60 ± 21.04	80.69 ± 8.81	531.06 ± 35.13
	WS71	6.54 ± 0.08	17.19 ± 0.10	1.65 ± 0.18	18.66 ± 0.96	3.91 ± 1.34	305.97 ± 27.26	72.56 ± 1.78	426.48 ± 31.70
	WS72	11.16 ± 0.24	16.36 ± 1.22	4.62 ± 0.48	28.51 ± 1.25	9.84 ± 3.21	581.83 ± 40.42	97.13 ± 4.56	749.44 ± 51.38
	WS73	2.88 ± 0.13	10.83 ± 0.32	4.72 ± 1.51	30.06 ± 0.39	12.45 ± 3.47	292.07 ± 28.02	76.03 ± 2.51	429.03 ± 36.35
	WS74	9.94 ± 5.21	13.61 ± 0.96	-0.11 ± 0.16	29.41 ± 1.71	7.61 ± 6.01	686.38 ± 32.75	126.40 ± 12.48	873.24 ± 59.28
	WS75	3.29 ± 0.13	10.93 ± 0.42	2.41 ± 0.49	33.63 ± 0.47	4.80 ± 0.43	452.24 ± 29.04	90.48 ± 11.34	597.79 ± 42.31
	WS76	14.10 ± 0.07	14.16 ± 0.68	7.46 ± 0.82	30.43 ± 2.78	6.24 ± 1.22	541.15 ± 24.90	97.94 ± 2.30	711.48 ± 32.77
	WS77	2.13 ± 0.15	11.33 ± 0.67	3.38 ± 0.19	43.32 ± 1.29	8.54 ± 0.79	304.42 ± 45.03	78.93 ± 9.35	452.04 ± 57.48
	WS78	2.50 ± 0.12	8.16 ± 0.18	0.48 ± 0.25	43.42 ± 0.87	10.13 ± 3.27	466.44 ± 27.62	98.22 ± 14.23	629.35 ± 46.55
	WS79	3.91 ± 0.04	12.59 ± 0.08	1.31 ± 0.30	27.26 ± 0.57	6.31 ± 3.68	431.67 ± 23.67	79.59 ± 11.16	562.65 ± 39.49
	WS80	3.71 ± 0.19	11.10 ± 0.25	5.06 ± 0.30	47.12 ± 0.80	8.62 ± 0.41	301.20 ± 20.37	75.28 ± 2.23	452.10 ± 24.55
	WS81	1.47 ± 0.42	8.38 ± 0.15	1.05 ± 0.05	33.53 ± 0.79	9.34 ± 0.11	518.96 ± 28.74	99.52 ± 12.11	672.24 ± 42.37
	WS82	5.02 ± 0.16	9.40 ± 0.46	1.55 ± 0.12	25.94 ± 0.94	8.34 ± 2.66	219.84 ± 190.47	86.35 ± 6.56	356.45 ± 201.36
	WS83	5.10 ± 0.17	16.55 ± 0.38	3.80 ± 2.22	27.04 ± 0.71	9.28 ± 0.87	319.58 ± 24.37	51.83 ± 8.12	433.19 ± 36.85
	WS84	4.97 ± 3.19	9.01 ± 1.33	2.50 ± 3.88	25.47 ± 0.97	9.87 ± 0.52	322.80 ± 36.83	87.87 ± 4.65	462.50 ± 51.37
	WS85	7.08 ± 2.74	11.22 ± 0.22	3.86 ± 3.21	21.74 ± 0.52	12.16 ± 0.80	351.35 ± 11.36	66.75 ± 3.57	474.16 ± 22.43
	WS86	4.46 ± 1.34	10.07 ± 0.13	2.52 ± 2.24	29.74 ± 0.63	8.60 ± 1.39	294.76 ± 39.32	71.77 ± 1.85	421.93 ± 46.89
Geumkang (commercial; WS87)		3.53 ± 0.06	7.28 ± 1.32	6.42 ± 2.39	44.13 ± 2.13	9.17 ± 3.05	209.00 ± 9.87	67.18 ± 4.58	346.71 ± 23.40
	WS88	2.83 ± 0.07	8.34 ± 0.55	5.68 ± 0.72	37.39 ± 1.96	10.32 ± 8.79	196.84 ± 21.90	60.47 ± 5.12	321.87 ± 39.11
	WS89	1.38 ± 1.31	13.49 ± 11.69	2.63 ± 2.55	28.63 ± 24.79	3.26 ± 3.07	199.37 ± 173.05	45.40 ± 39.52	294.16 ± 255.98
	WS90	3.05 ± 0.42	8.48 ± 1.56	5.86 ± 0.54	34.82 ± 3.87	10.94 ± 6.37	173.55 ± 152.08	72.57 ± 6.92	309.27 ± 171.76
Cheongwoo (commercial; WS91)		4.81 ± 0.85	10.43 ± 0.86	6.28 ± 0.73	26.14 ± 1.18	10.74 ± 2.66	217.67 ± 17.69	67.29 ± 2.73	343.36 ± 26.70

^a^ All values are presented as the mean ± standard deviation (SD) of triplicate measurements. ^b^ Eicosanol and heneicosanol were not detected. ^c^ C22-OH, docosanol; C23-OH, tricosanol; C24-OH, tetracosanol; C26-OH, hexacosanol; C27-OH, heptacosanol; C28-OH, octacosanol; C30-OH, triacontanol.

**Table 3:** Individual and total policosanol contents in the sprout samples of the top 10 wheat mutant lines cultivated under blue (440 nm) LED light irradiation.

Cultivars	Mutant Lines	Policosanol Content (mg/100 g Dry Weight) ^a,b^							
		C22-OH ^c^	C23-OH ^c^	C24-OH ^c^	C26-OH ^c^	C27-OH ^c^	C28-OH ^c^	C30-OH ^c^	Total PC
Woori-mil × D-7 (original; WS01)		ND ^d^	3.89 ± 0.33	0.99 ± 0.85	21.62 ± 2.09	3.77 ± 0.43	394.04 ± 9.94	26.74 ± 1.91	451.06 ± 15.55
	WS37	ND	3.55 ± 0.40	ND	13.76 ± 1.13	2.68 ± 0.37	297.20 ± 11.32	22.03 ± 1.96	339.21 ± 15.12
	WS49	ND	6.91 ± 0.89	4.00 ± 0.31	53.91 ± 5.31	5.39 ± 0.62	448.52 ± 23.34	18.20 ± 1.70	536.94 ± 32.17
	WS57	ND	6.49 ± 0.49	ND	41.48 ± 4.15	3.98 ± 0.56	393.50 ± 30.34	25.17 ± 2.83	470.63 ± 38.37
	WS69	4.31 ± 0.54	6.88 ± 0.71	3.12 ± 0.50	15.10 ± 1.28	3.19 ± 0.16	360.84 ± 21.47	28.90 ± 2.68	422.35 ± 27.34
	WS72	0.99 ± 0.28	5.24 ± 0.60	1.64 ± 0.36	11.06 ± 1.22	2.59 ± 0.43	292.60 ± 17.32	20.20 ± 2.30	334.33 ± 22.51
	WS74	2.76 ± 0.87	9.03 ± 0.80	3.45 ± 1.60	18.43 ± 1.61	4.25 ± 0.58	496.20 ± 27.75	33.42 ± 2.61	567.54 ± 35.81
	WS75	2.14 ± 0.23	6.59 ± 0.87	3.26 ± 0.32	17.99 ± 1.45	2.38 ± 0.39	353.03 ± 22.60	24.71 ± 2.37	410.01 ± 28.24
	WS76	5.36 ± 2.18	4.68 ± 0.76	5.54 ± 1.28	25.32 ± 2.73	3.74 ± 0.48	445.55 ± 21.85	34.98 ± 5.27	525.16 ± 34.53
	WS78	ND	3.61 ± 0.26	ND	27.37 ± 2.73	3.89 ± 0.15	363.45 ± 21.40	27.21 ± 4.76	425.54 ± 29.29
	WS81	1.32 ± 0.49	4.79 ± 0.60	2.67 ± 0.12	19.45 ± 1.95	3.12 ± 0.11	377.09 ± 15.30	27.23 ± 2.36	435.68 ± 20.92

^a^ All values are presented as the mean ± SD of triplicate determinations. ^b^ Eicosanol and heneicosanol were not detected. ^c^ C22-OH, docosanol; C23-OH, tricosanol; C24-OH, tetracosanol; C26-OH, hexacosanol; C27-OH, heptacosanol; C28-OH, octacosanol; C30-OH, triacontanol. ^d^ Not detected.

**Table 4:** Individual and total policosanol contents in the sprout samples of the top 10 wheat mutant lines cultivated under green (520 nm) LED light irradiation.

Cultivars	Mutant Lines	Policosanol Content (mg/100 g Dry Weight) ^a,b^							
		C22-OH ^c^	C23-OH ^c^	C24-OH ^c^	C26-OH ^c^	C27-OH ^c^	C28-OH ^c^	C30-OH ^c^	Total PC
Woori-mil × D-7 (original; WS01)		2.51 ± 0.56	8.77 ± 1.22	3.84 ± 0.77	9.79 ± 1.86	1.47 ± 0.32	169.31 ± 8.92	9.40 ± 0.94	205.08 ± 14.59
	WS37	ND ^d^	10.60 ± 0.97	1.41 ± 0.22	8.36 ± 0.56	1.62 ± 0.10	208.41 ± 8.77	14.69 ± 0.83	245.10 ± 11.44
	WS49	2.21 ± 0.22	10.5 ± 1.6	4.82 ± 1.02	36.18 ± 3.83	3.33 ± 0.44	309.97 ± 11.27	12.84 ± 1.51	379.82 ± 19.88
	WS57	1.06 ± 0.12	12.1 ± 1.3	2.77 ± 0.18	20.97 ± 1.64	2.03 ± 0.31	202.37 ± 9.73	11.01 ± 0.87	252.31 ± 14.19
	WS69	ND	8.2 ± 1.2	ND	8.31 ± 2.94	ND	289.79 ± 27.30	12.70 ± 3.89	318.96 ± 35.30
	WS72	3.25 ± 1.00	15.4 ± 1.2	2.57 ± 0.15	11.37 ± 1.11	1.96 ± 0.29	256.28 ± 23.17	18.87 ± 1.92	309.73 ± 28.85
	WS74	ND	9.0 ± 1.2	4.65 ± 1.21	ND	ND	268.88 ± 45.96	14.14 ± 1.56	296.70 ± 49.90
	WS75	7.30 ± 1.39	13.9 ± 1.1	4.03 ± 0.80	16.29 ± 1.91	ND	270.53 ± 13.58	19.82 ± 1.34	331.87 ± 20.11
	WS76	2.45 ± 0.12	14.5 ± 1.6	3.95 ± 1.14	15.95 ± 1.42	1.78 ± 0.36	262.53 ± 13.74	17.30 ± 2.31	318.48 ± 20.66
	WS78	3.79 ± 0.21	7.8 ± 0.6	2.32 ± 0.57	15.24 ± 0.88	2.18 ± 0.36	216.93 ± 9.61	13.93 ± 0.66	262.18 ± 12.93
	WS81	1.22 ± 0.02	11.8 ± 1.4	2.01 ± 0.23	5.91 ± 1.22	ND	145.81 ± 10.33	8.81 ± 1.29	175.54 ± 14.54

^a^ All values are presented as the mean ± SD of triplicate determinations. ^b^ Eicosanol and heneicosanol were not detected. ^c^ C22-OH, docosanol; C23-OH, tricosanol; C24-OH, tetracosanol; C26-OH, hexacosanol; C27-OH, heptacosanol; C28-OH, octacosanol; C30-OH, triacontanol. ^d^ Not detected.

**Table 5:** Individual and total policosanol contents in the sprout samples of the top 10 wheat mutant lines cultivated under red (660 nm) LED light irradiation.

Cultivars	Mutant Lines	Policosanol Content (mg/100 g Dry Weight) ^a,b^							
		C22-OH ^c^	C23-OH ^c^	C24-OH ^c^	C26-OH ^c^	C27-OH ^c^	C28-OH ^c^	C30-OH ^c^	Total PC
Woori-mil × D-7 (original; WS01)		0.60 ± 0.16	15.31 ± 1.97	2.27 ± 0.64	17.11 ± 5.10	3.03 ± 0.77	324.80 ± 25.77	19.50 ± 3.32	382.61 ± 37.74
	WS37	ND ^d^	9.33 ± 0.88	1.36 ± 0.13	10.01 ± 1.04	1.24 ± 0.16	234.17 ± 9.81	16.11 ± 0.22	272.22 ± 12.23
	WS49	0.63 ± 0.03	10.31 ± 1.03	3.96 ± 0.22	40.08 ± 3.71	3.46 ± 0.34	338.52 ± 14.53	13.31 ± 1.11	410.27 ± 20.97
	WS57	0.82 ± 0.15	10.67 ± 0.81	2.20 ± 0.20	22.13 ± 2.44	2.31 ± 0.17	246.80 ± 15.17	13.24 ± 1.13	298.16 ± 20.06
	WS69	ND	12.47 ± 1.58	1.75 ± 0.33	10.33 ± 2.33	2.52 ± 1.14	270.68 ± 17.79	14.27 ± 0.91	312.02 ± 24.09
	WS72	ND	22.27 ± 2.06	2.93 ± 0.47	20.45 ± 1.28	2.96 ± 0.33	455.58 ± 25.94	29.88 ± 3.73	534.07 ± 33.79
	WS74	ND	9.17 ± 0.77	ND	4.19 ± 2.24	4.74 ± 2.36	268.94 ± 50.45	24.28 ± 19.81	311.32 ± 75.64
	WS75	1.82 ± 0.12	15.81 ± 1.83	3.59 ± 0.10	14.79 ± 1.18	2.08 ± 0.23	280.17 ± 15.95	17.61 ± 2.65	335.86 ± 22.05
	WS76	1.67 ± 0.07	14.55 ± 1.47	3.30 ± 0.58	14.30 ± 0.82	2.14 ± 0.66	292.10 ± 18.02	16.59 ± 1.57	344.64 ± 23.18
	WS78	ND	7.69 ± 0.62	2.56 ± 0.30	29.98 ± 2.74	4.20 ± 0.81	333.04 ± 13.82	21.31 ± 1.66	398.78 ± 19.94
	WS81	0.66 ± 0.09	9.14 ± 0.81	2.32 ± 0.17	11.14 ± 1.52	1.44 ± 0.12	215.82 ± 12.17	12.54 ± 1.21	253.06 ± 16.10

^a^ All values are presented as the mean ± SD of triplicate determinations. ^b^ Eicosanol and heneicosanol were not detected. ^c^ C22-OH, docosanol; C23-OH, tricosanol; C24-OH, tetracosanol; C26-OH, hexacosanol; C27-OH, heptacosanol; C28-OH, octacosanol; C30-OH, triacontanol. ^d^ Not detected.

## Data Availability

Not applicable.

## Supplementary Materials

The following supporting information can be downloaded at https://www.mdpi.com/article/10.3390/plants12193377/s1, Figure S1: Chromatogram and mass spectrum of policosanols (20 ppm) using a GCMS-QP2010 Ultra (Shimadzu, Kyoto, Japan). Figure S2: Representative chromatogram of the sprout extract of the original variety and the selected 10 mutant lines of wheat, which were cultivated in a growth camber exposed to a white LED light. Figure S3: Chromatogram and mass spectrum of policosanols (20 ppm) using a GCMS-QP2020 NX (Shimadzu). Figure S4: Representative chromatogram of the sprout extract of the original variety and the selected 10 mutant lines of wheat, which were cultivated in a growth camber exposed to a blue LED light. Figure S5: Representative chromatogram of the sprout extract of the original variety and the selected 10 mutant lines of wheat, which were cultivated in a growth camber exposed to a green LED light. Figure S6: Representative chromatogram of the sprout extract of the original variety and the selected 10 mutant lines of wheat, which were cultivated in a growth camber exposed to a red LED light.
