# Supplementary material for: Comparison of Policosanol Profiles of the Sprouts of Wheat Mutant Lines and the Effect of Differential LED Lights on Selected Lines
Source: Plants (Basel). 2023 Sep 25;12(19):3377. doi: 10.3390/plants12193377 (PMC10574449; doi:10.3390/plants12193377)
Supplement: Supplementary file 1 [file plants-12-03377-s001.zip › plants-2594942-supplementary.pdf]

Supporting Information

Comparison of Policosanol Profiles of the Sprouts of Wheat Mutant Lines and the Effect of Differential LED Lights on Selected Lines

Ah-Reum Han *, Euna Choi, Jisu Park, Sang Hee Jo, Min Jeong Hong, Jin-Baek Kim, Ga-Hee Ryoo, Chang Hyun Jin

Advanced Radiation Technology Institute, Korea Atomic Energy Research Institute, Jeongeup-si, Jeollabuk-do 56212, Republic of Korea; euna3388@kaeri.re.kr (E.C.); parkjs94@kaeri.re.kr (J.P.); shjo@kaeri.re.kr (S.H.J.); hongmj@kaeri.re.kr (M.J.H.); jbkim74@kaeri.re.kr (J.-B.K.); ghryoo@kaeri.re.kr (G.H.R.); chjin@kaeri.re.kr (C.H.J.)

* Correspondence: arhan@kaeri.re.kr (A.-R.H.); Tel.: +82-63-570-3167 (A.-R.H.)

CONTENTS

Figure S1. Chromatogram and mass spectrum of policosanols (20 ppm) using a GCMS-QP2010 Ultra (Shimadzu, Kyoto, Japan).

Figure S2. Representative chromatogram of the sprout extract of the original variety and the selected 10 mutant lines of wheat which were cultivated in a growth camber exposed to a white LED light.

Figure S3. Chromatogram and mass spectrum of policosanols (20 ppm) using a GCMS-QP2020 NX (Shimadzu).

Figure S4. Representative chromatogram of the sprout extract of the original variety and the selected 10 mutant lines of wheat which were cultivated in a growth camber exposed to a blue LED light.

Figure S5. Representative chromatogram of the sprout extract of the original variety and the selected 10 mutant lines of wheat which were cultivated in a growth camber exposed to a green LED light.

Figure S6. Representative chromatogram of the sprout extract of the original variety and the selected 10 mutant lines of wheat which were cultivated in a growth camber exposed to a red LED light.

Policosanols	Gas chromatogram	Mass spectrum
Eicosanol (C20-OH)	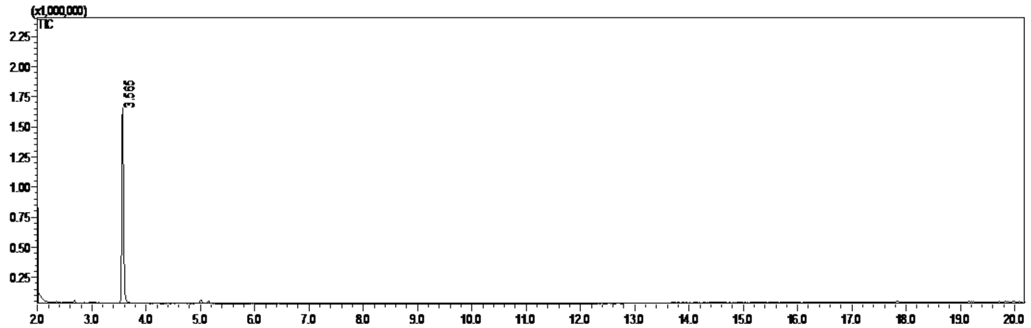	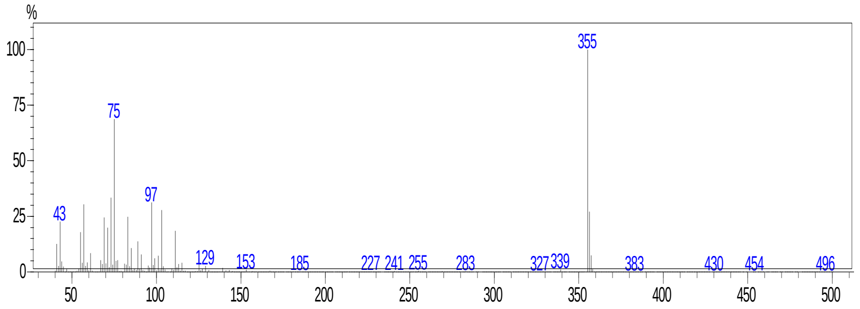
Heneicosanol (C21-OH)	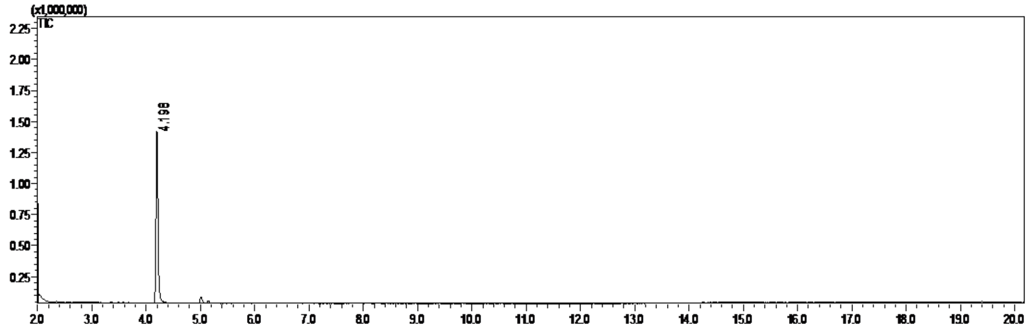	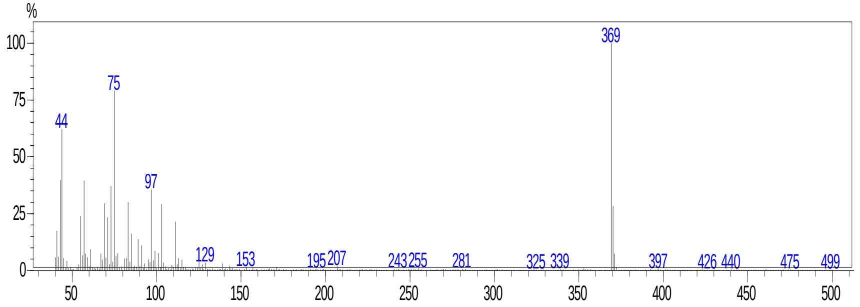
Docosanol (C22-OH)	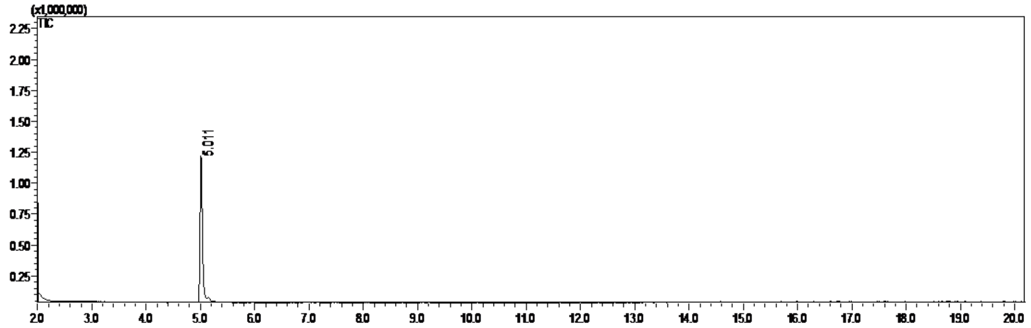	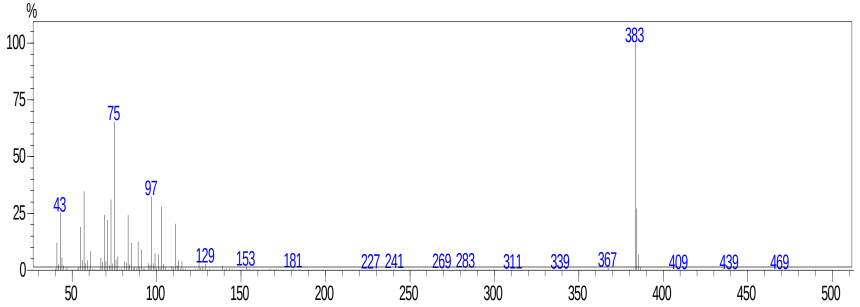

Figure S1. Chromatogram and mass spectrum of policosanols (20 ppm) using a GCMS-QP2010 Ultra (Shimadzu, Kyoto, Japan).

Policosanols	Gas chromatogram	Mass spectrum
Tricosanol (C23-OH)	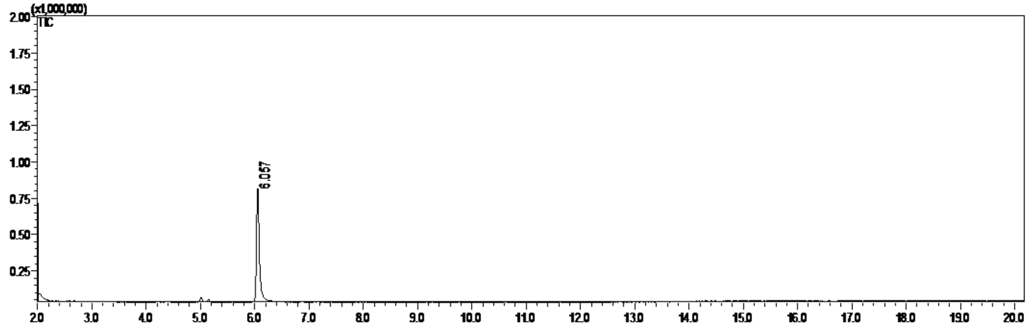	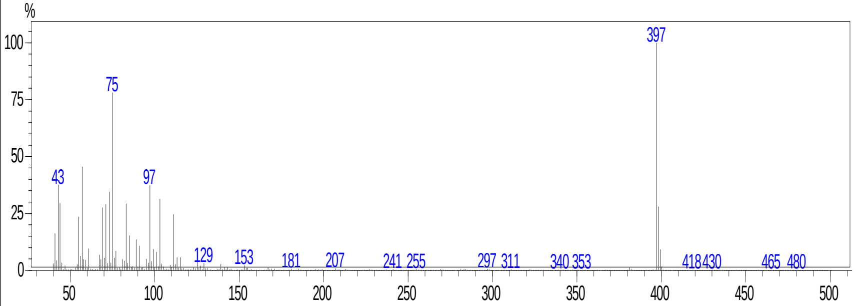
Tetracosanol (C24-OH)	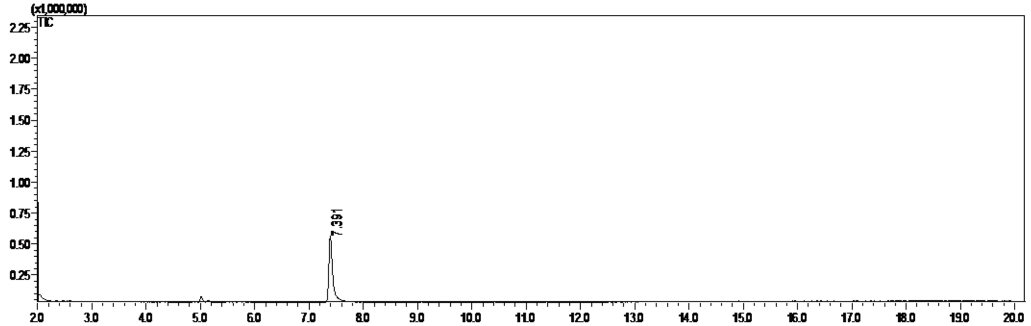	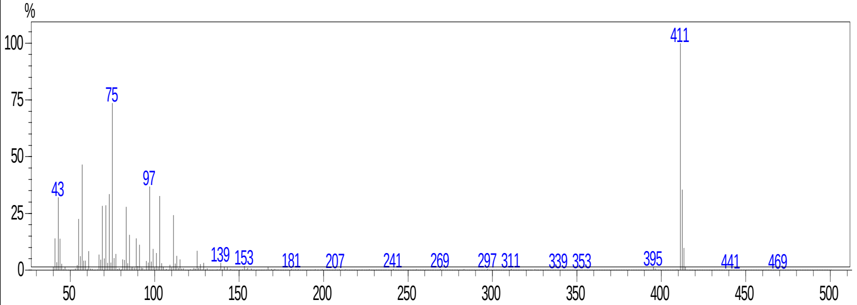
Hexacosanol (C26-OH)	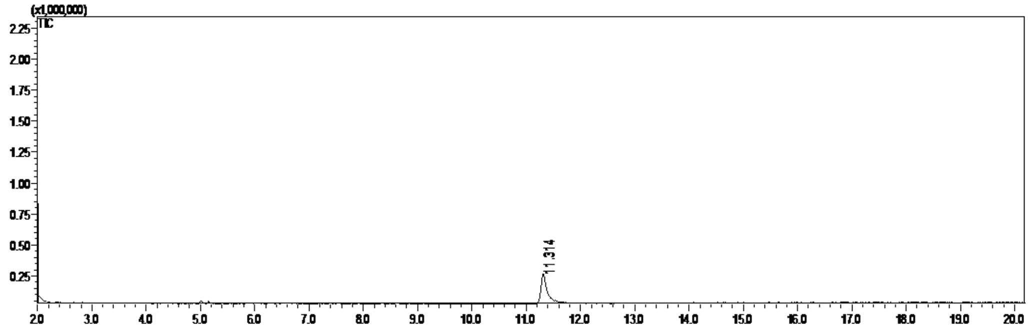	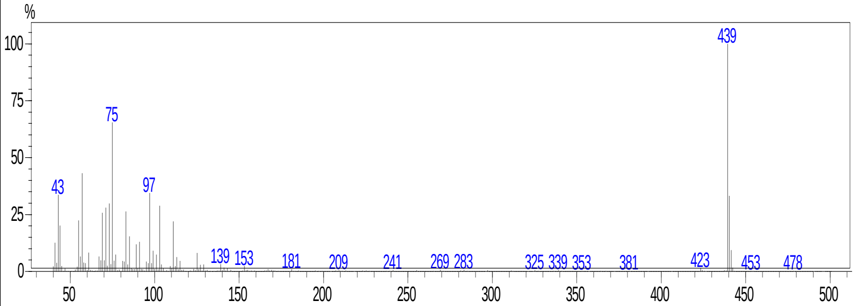

Figure S1. Chromatogram and mass spectrum of policosanols (20 ppm) using a GCMS-QP2010 Ultra (Shimadzu, Kyoto, Japan) (*cont.*)

<table>
<tr><th>Policosanols</th><th>Gas chromatogram</th><th>Mass spectrum</th></tr>
<tr><td>Heptacosanol (C27-OH)</td><td>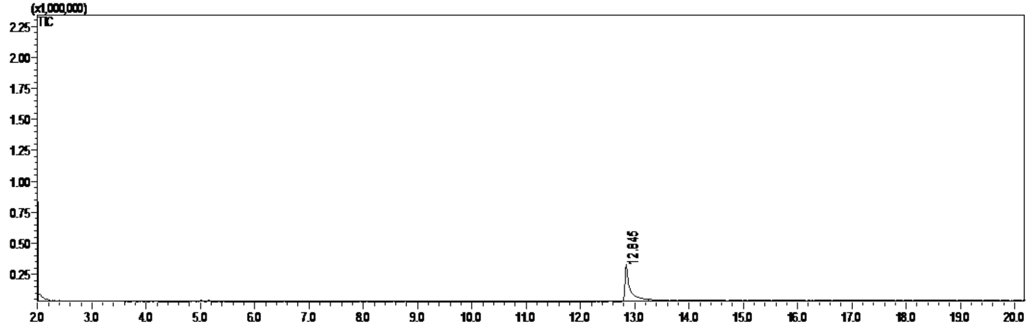
</td><td>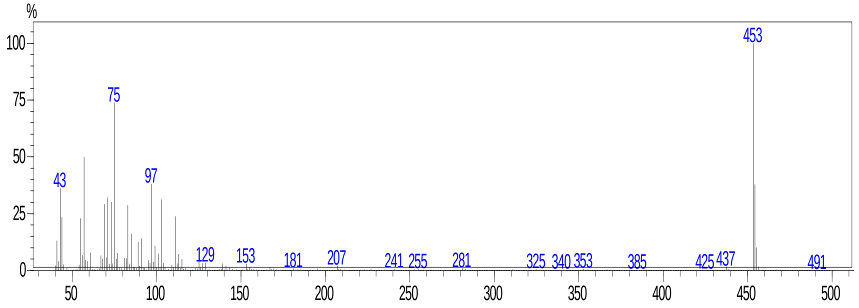
</td></tr>
<tr><td>Octacosanol (C28-OH)</td><td>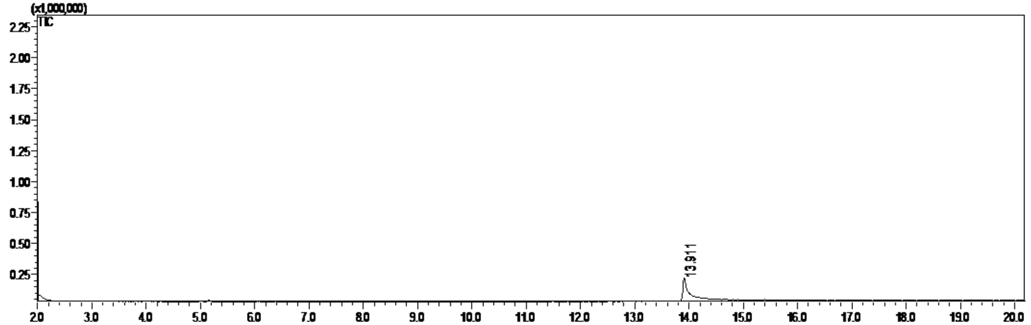
</td><td>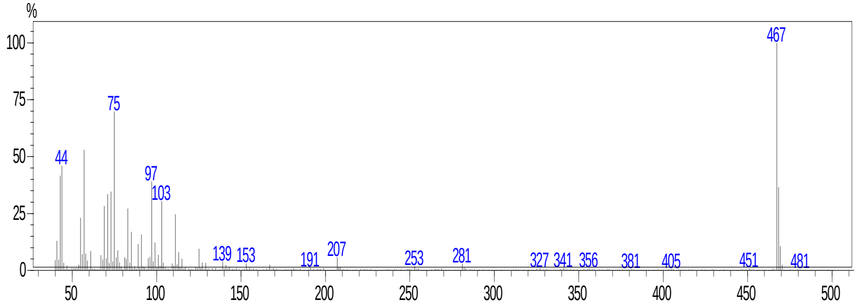
</td></tr>
<tr><td>Triacontanol (C30-OH)</td><td>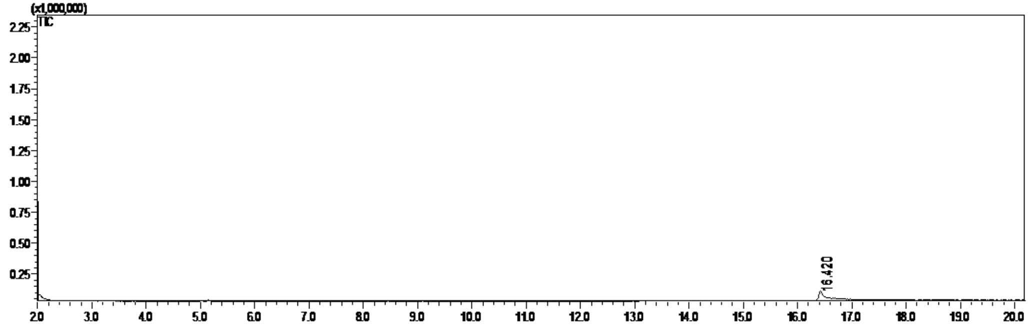
</td><td>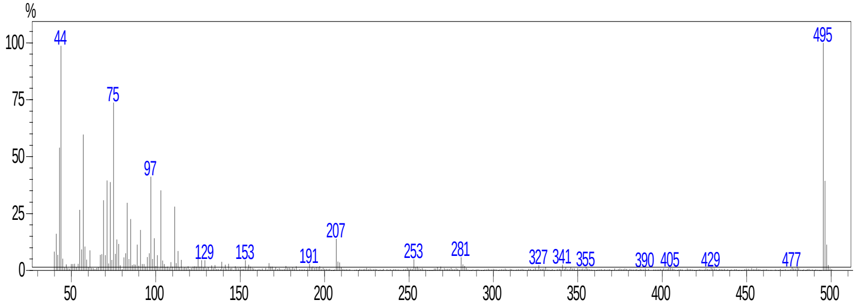
</td></tr>
</table>

Figure S1. Chromatogram and mass spectrum of policosanols (20 ppm) using a GCMS-QP2010 Ultra (Shimadzu, Kyoto, Japan) (*cont.*)

<table>
<tr><th>Wheat sprout samples</th><th>Gas chromatogram</th></tr>
<tr><td>WS01
Plot18</td><td>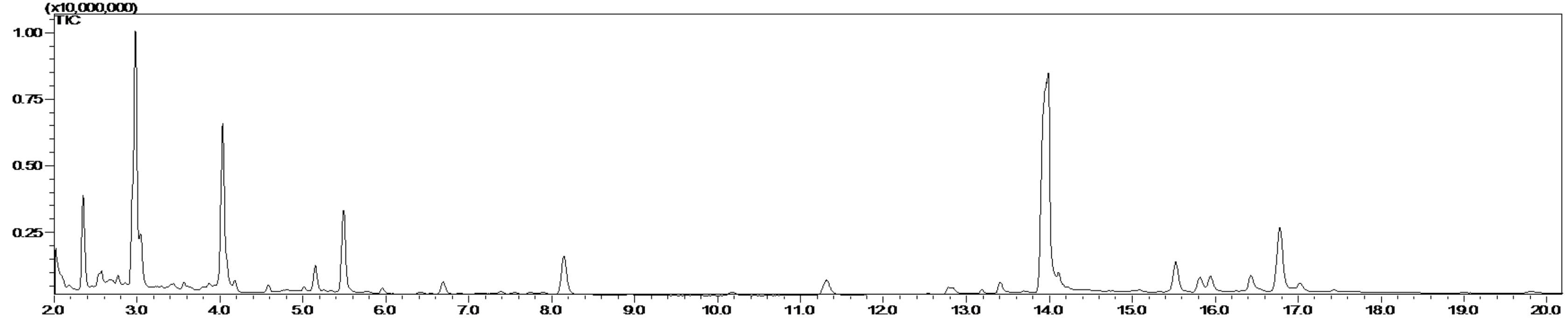</td></tr>
<tr><td>WS37
Plot46</td><td>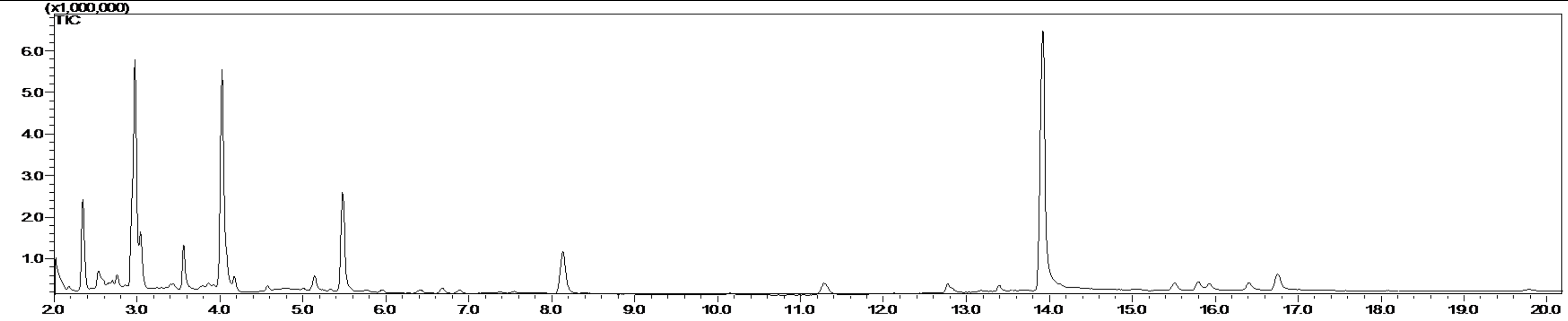</td></tr>
<tr><td>WS49
PlotCW263</td><td>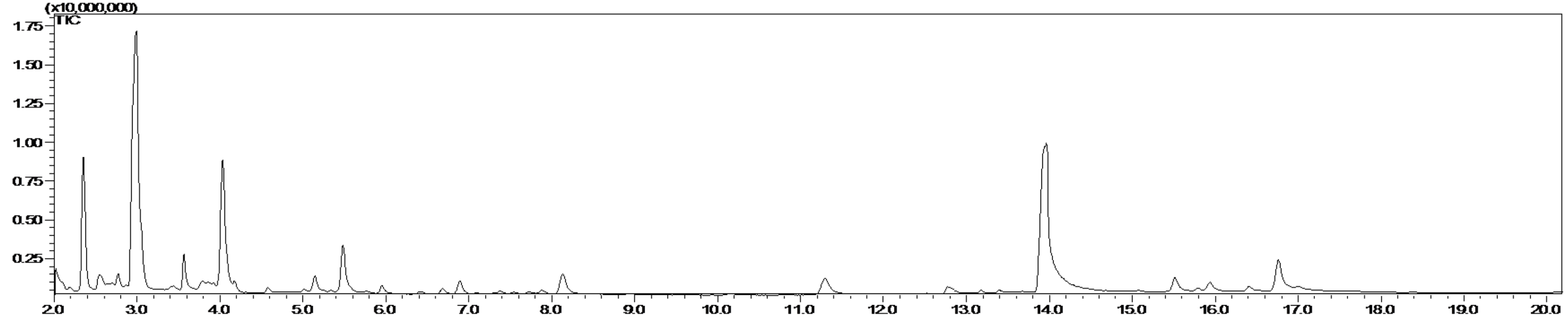</td></tr>
</table>

Figure S2. Representative chromatogram of the sprout extract of the original variety and the selected 10 mutant lines of wheat which were cultivated in a growth camber exposed to a white LED light.

<table>
<tr><th>Wheat sprout samples</th><th>Gas chromatogram</th></tr>
<tr><td>WS57
PlotCW1027</td><td>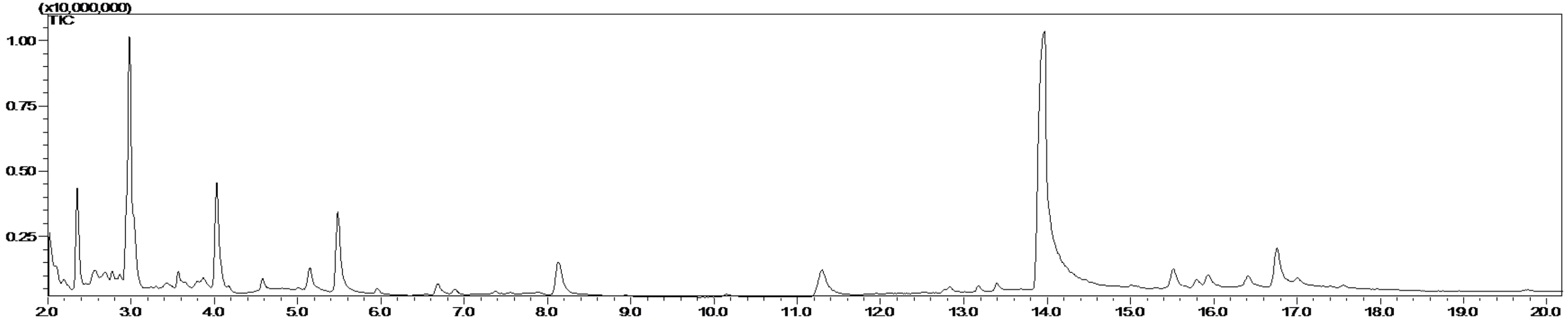
</td></tr>
<tr><td>WS69
Plot80</td><td>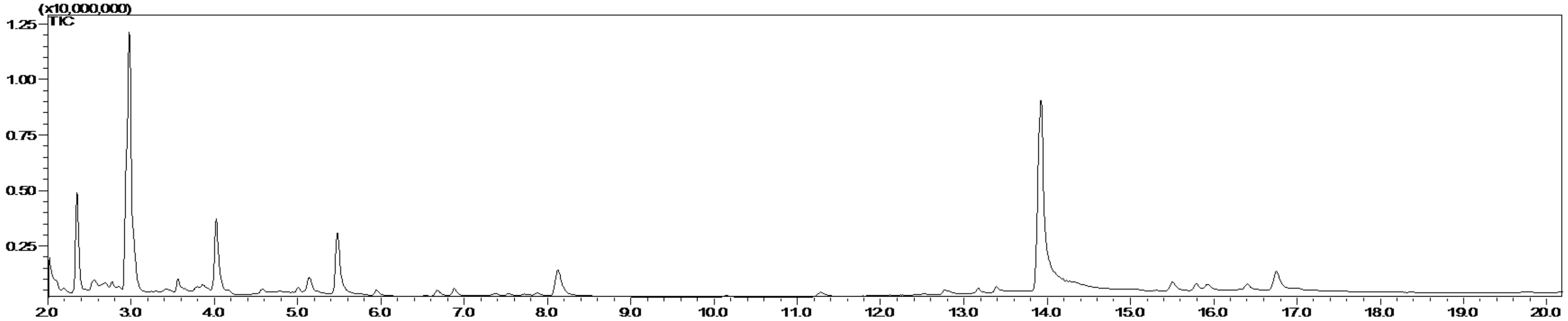
</td></tr>
<tr><td>WS72
Plot83</td><td>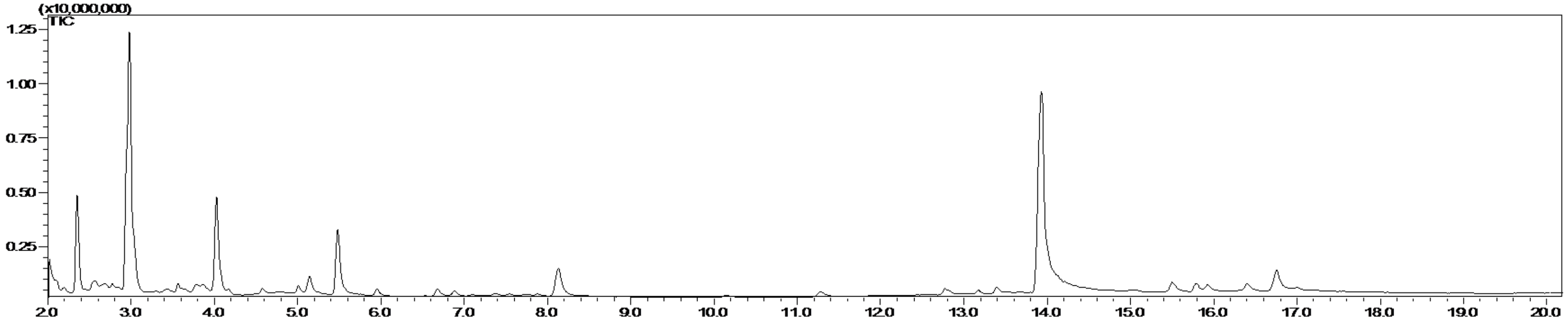
</td></tr>
</table>

Figure S2. Representative chromatogram of the sprout extract of the original variety and the selected 10 mutant lines of wheat which were cultivated in a growth camber exposed to a white LED light (*cont.*).

Wheat sprout samples	Gas chromatogram
WS74<br>Plot85	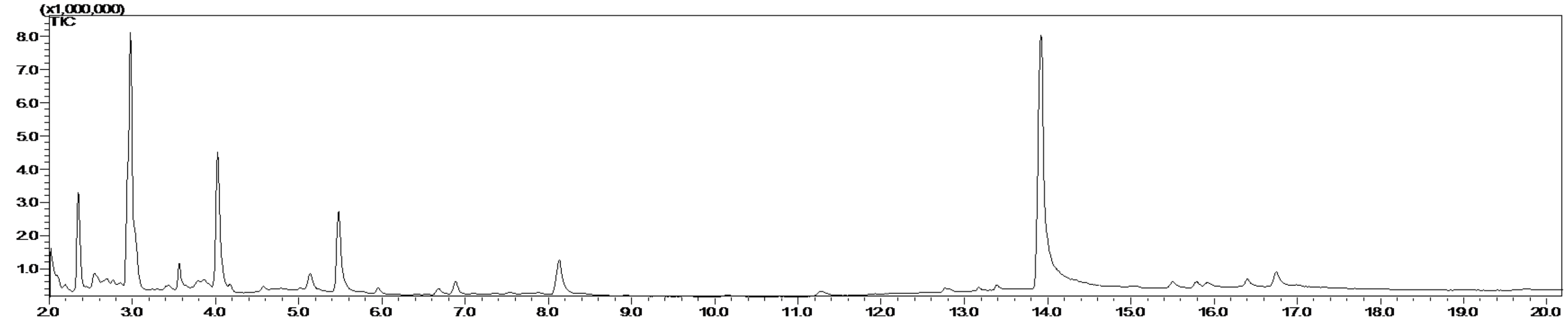
WS75<br>Plot86	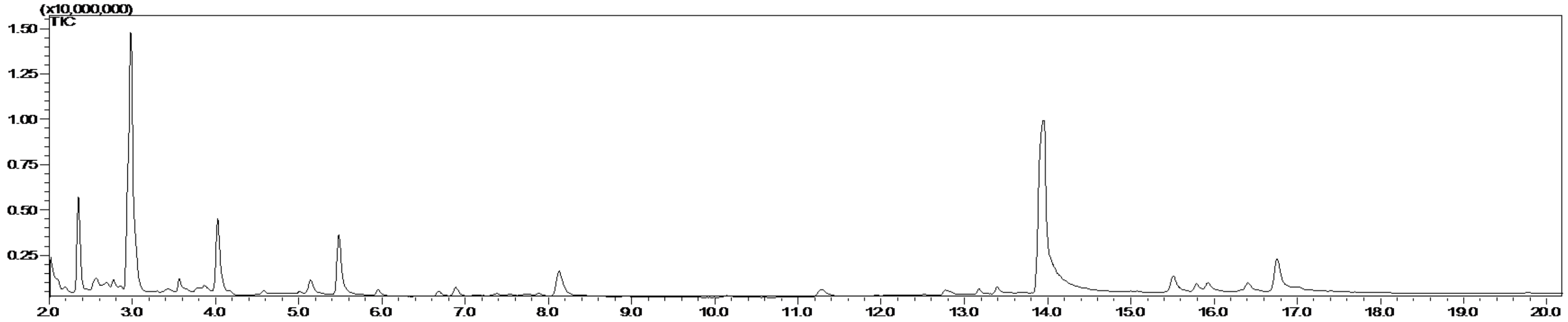
WS76<br>Plot87	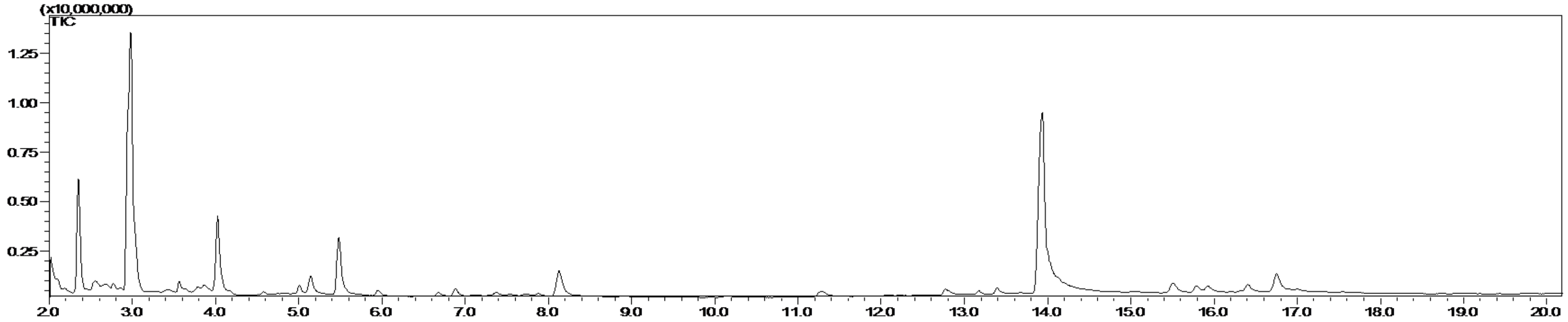

Figure S2. Representative chromatogram of the sprout extract of the original variety and the selected 10 mutant lines of wheat which were cultivated in a growth camber exposed to a white LED light (*cont.*).

Wheat sprout samples	Gas chromatogram
WS78<br>Plot89	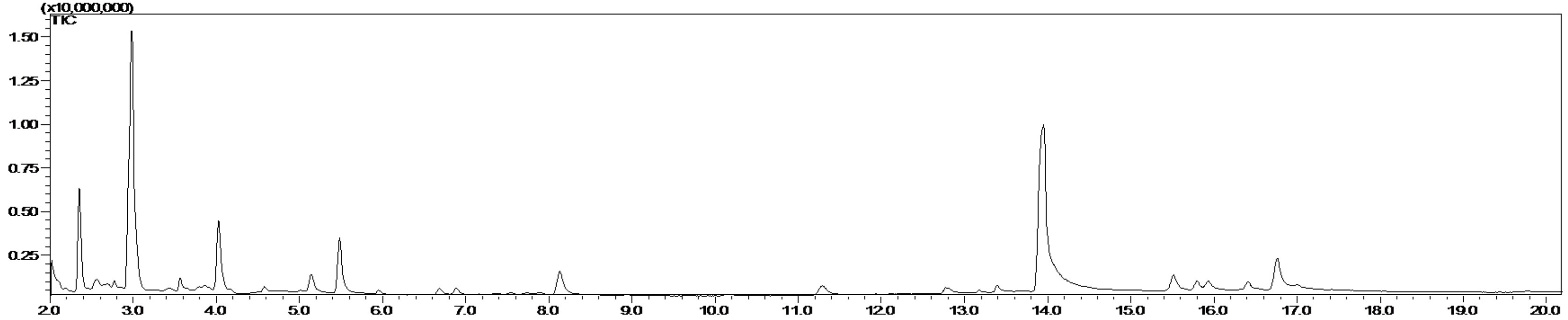
WS81<br>Plot92	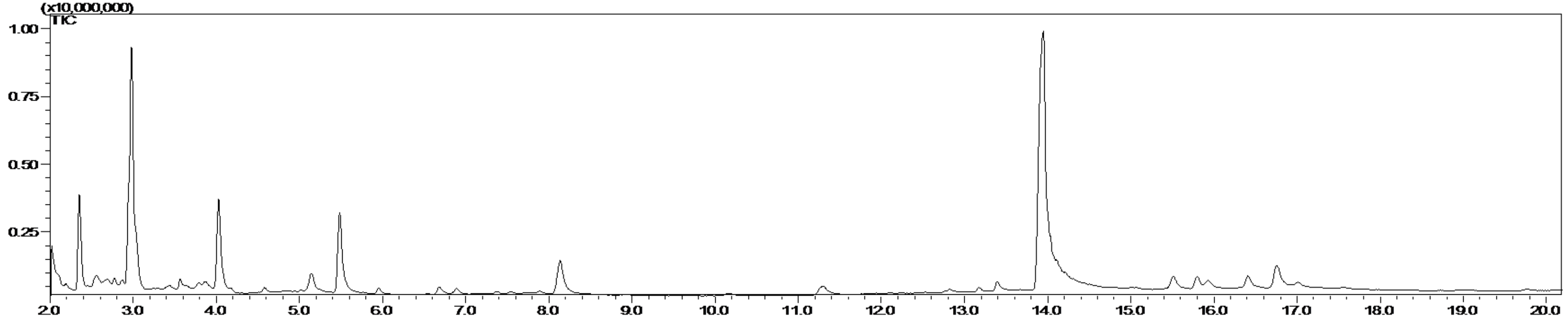

Figure S2. Representative chromatogram of the sprout extract of the original variety and the selected 10 mutant lines of wheat which were cultivated in a growth camber exposed to a white LED light (*cont.*).

<table>
<tr><th>Policosanols</th><th>Gas chromatogram</th><th>Mass spectrum</th></tr>
<tr><td>Eicosanol (C20-OH)</td><td>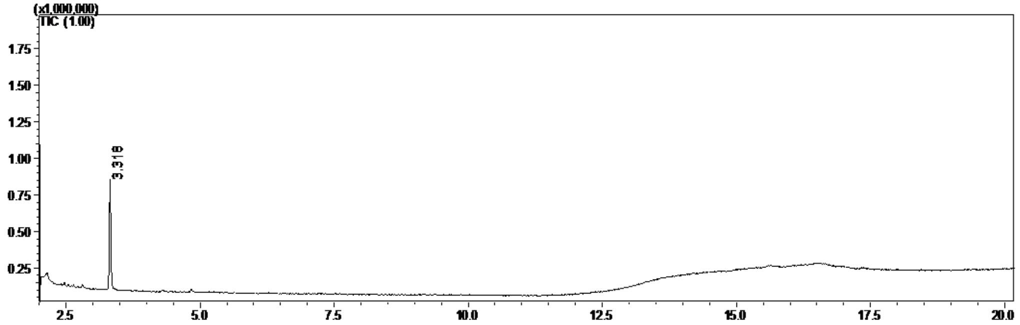
</td><td>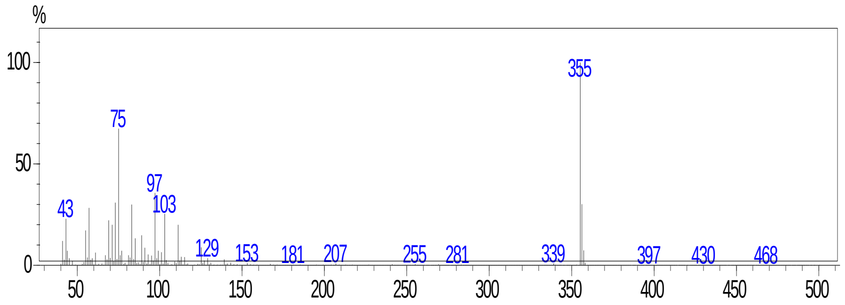
</td></tr>
<tr><td>Heneicosanol (C21-OH)</td><td>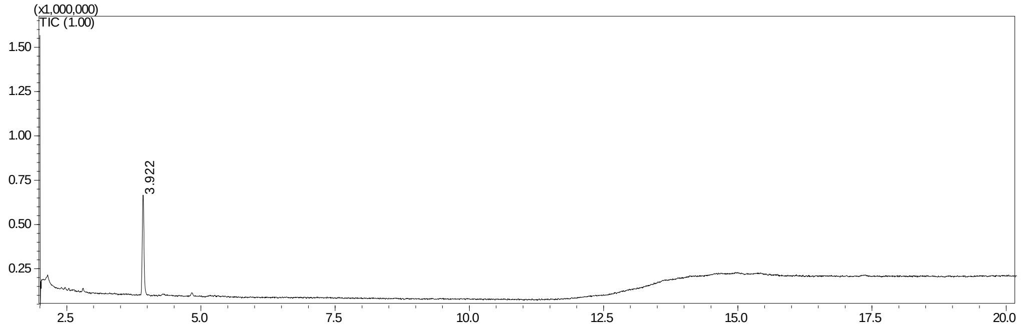
</td><td>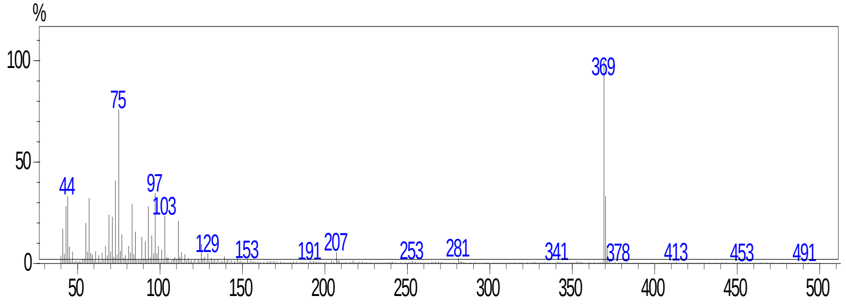
</td></tr>
<tr><td>Docosanol (C22-OH)</td><td>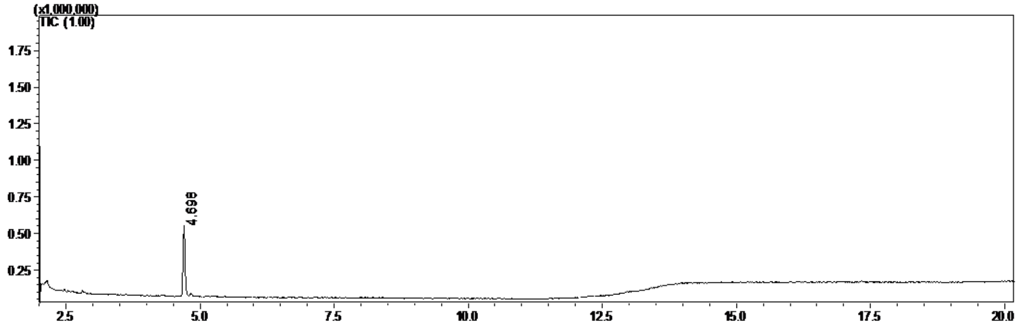
</td><td>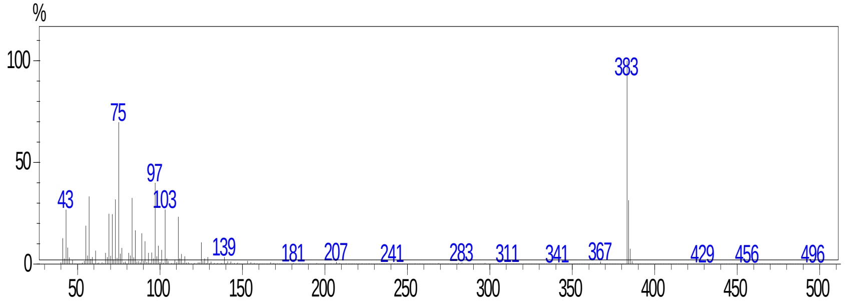
</td></tr>
</table>

Figure S3. Chromatogram and mass spectrum of policosanols (20 ppm) using a GCMS-QP2020 NX (Shimadzu).

Policosanols	Gas chromatogram	Mass spectrum
Tricosanol (C23-OH)	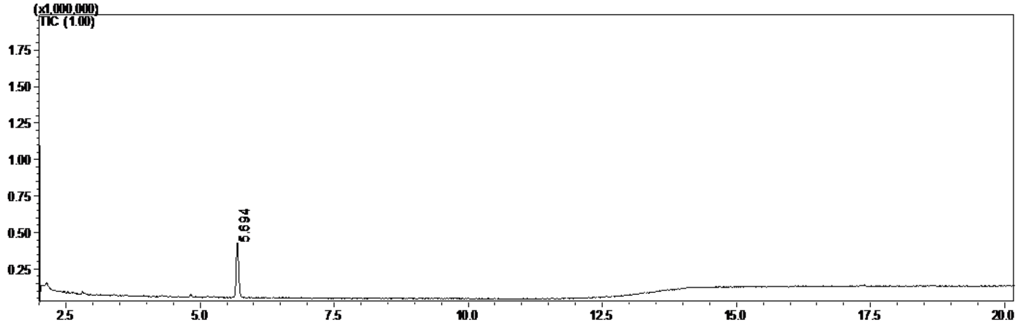	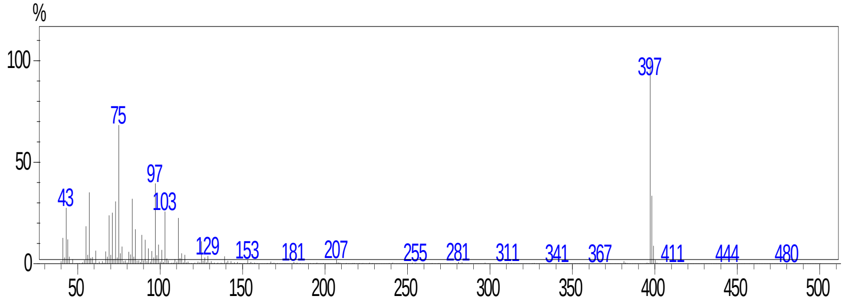
Tetracosanol (C24-OH)	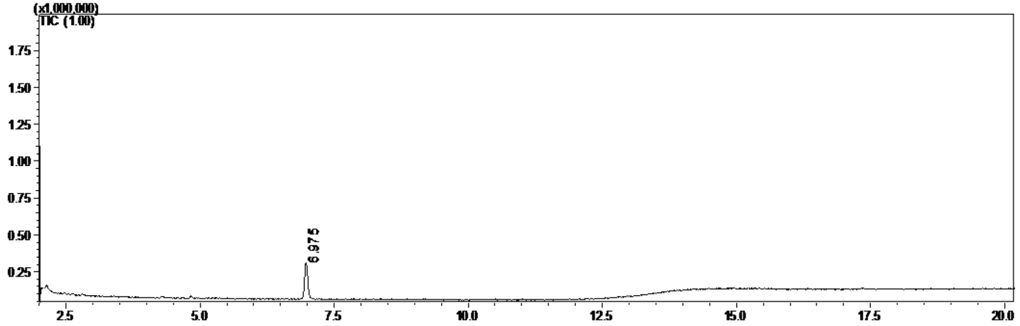	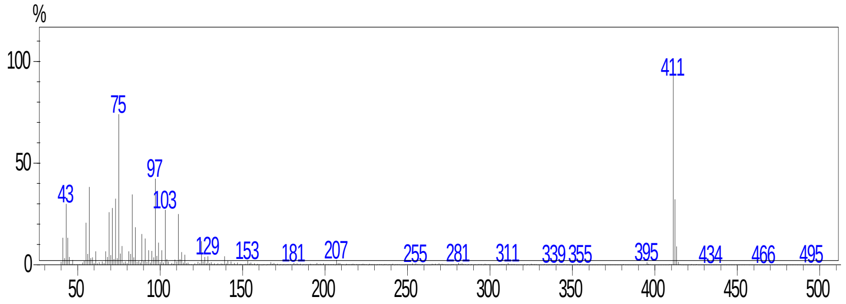
Hexacosanol (C26-OH)	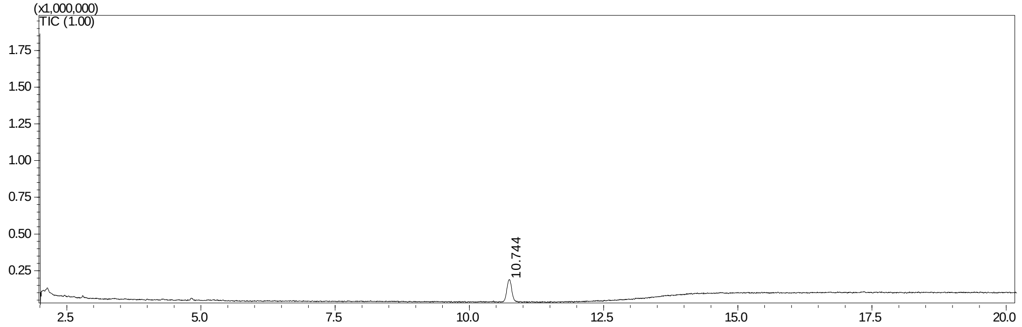	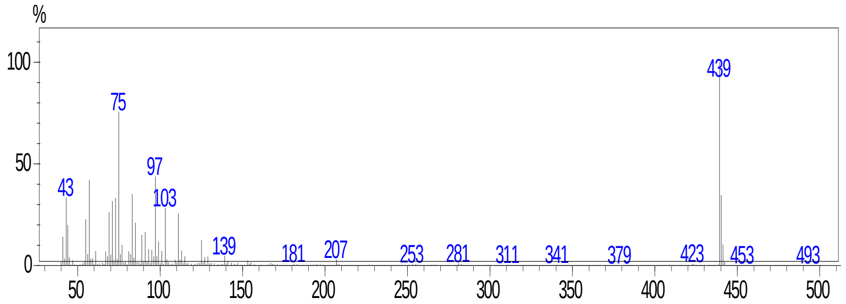

Figure S3. Chromatogram and mass spectrum of policosanols (20 ppm) using a GCMS-QP2020 NX (Shimadzu) (*cont.*)

Policosanols	Gas chromatogram	Mass spectrum
Heptacosanol (C27-OH)	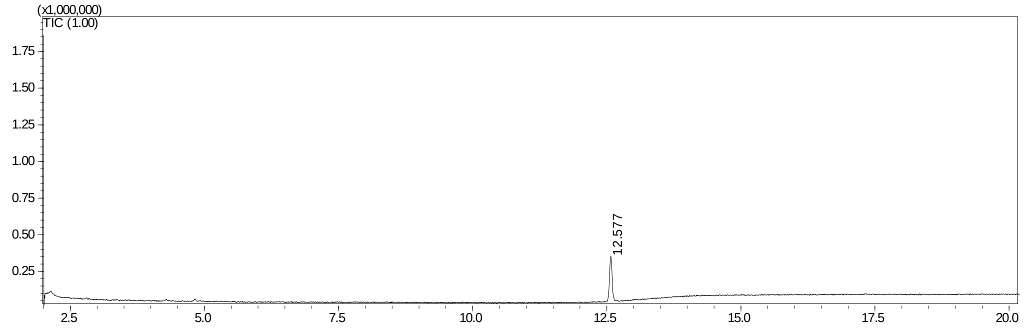	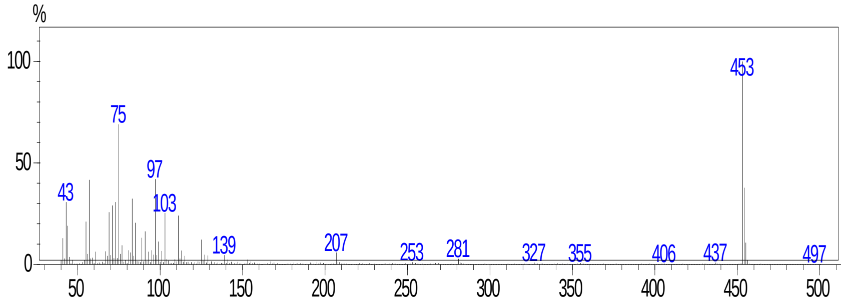
Octacosanol (C28-OH)	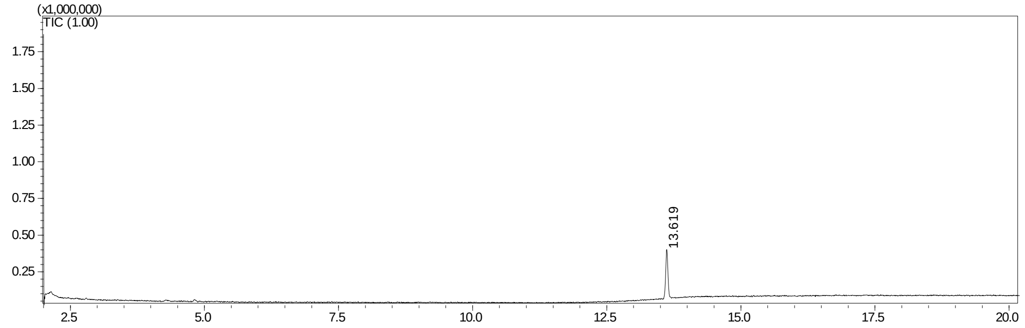	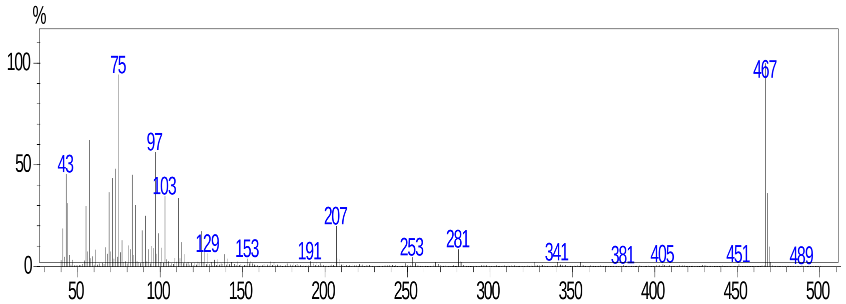
Triacontanol (C30-OH)	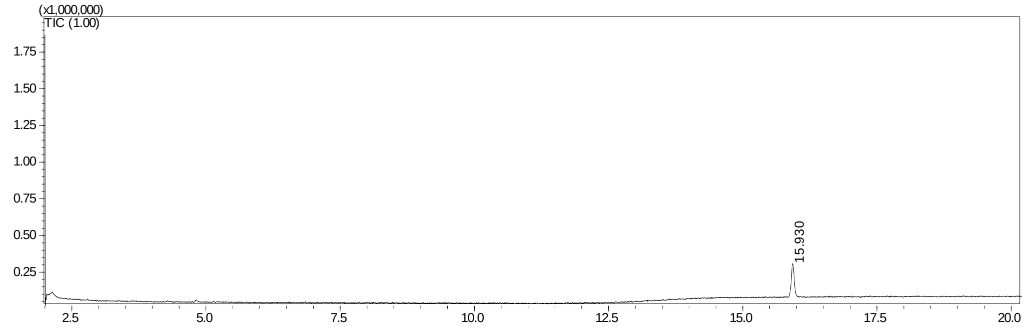	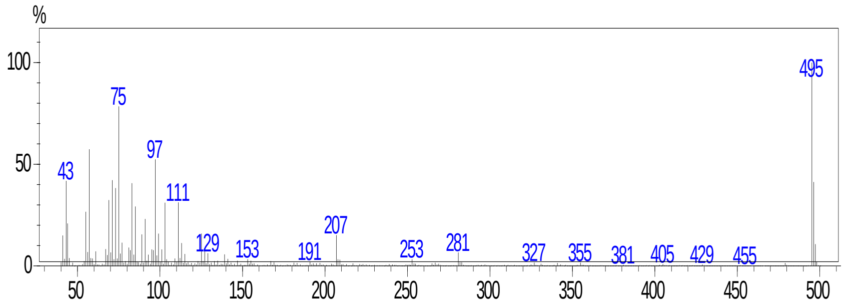

Figure S3. Chromatogram and mass spectrum of policosanols (20 ppm) using a GCMS-QP2020 NX (Shimadzu) (*cont.*)

Wheat sprout samples	Gas chromatogram
WS01<br>Plot18	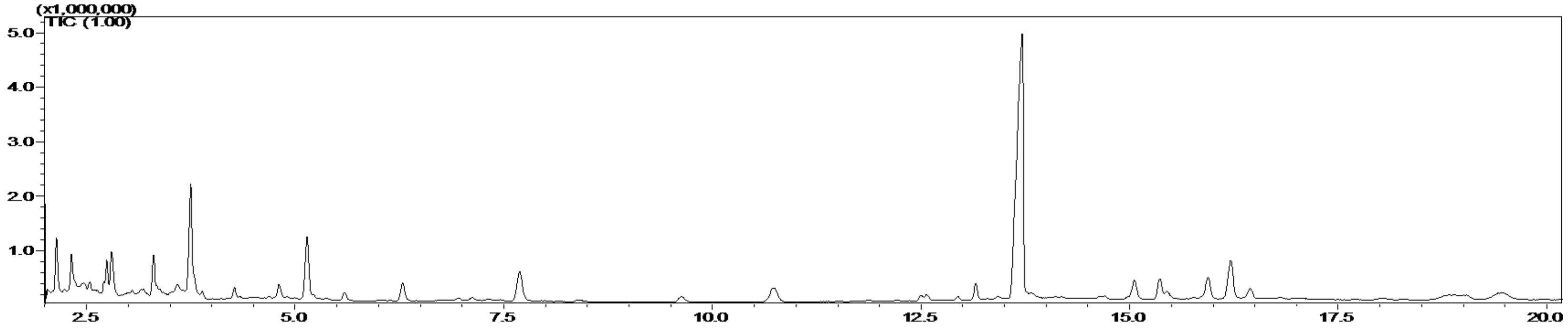
WS37<br>Plot46	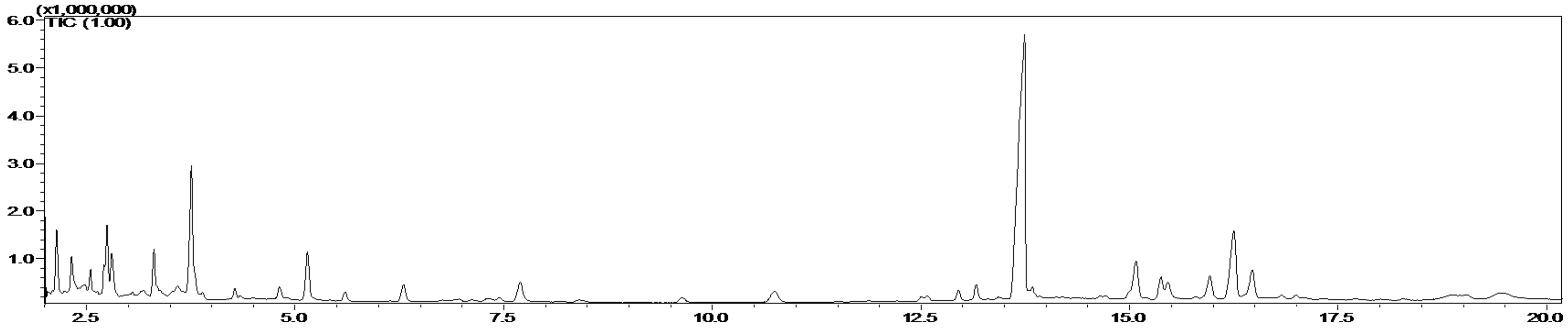
WS49<br>PlotCW263	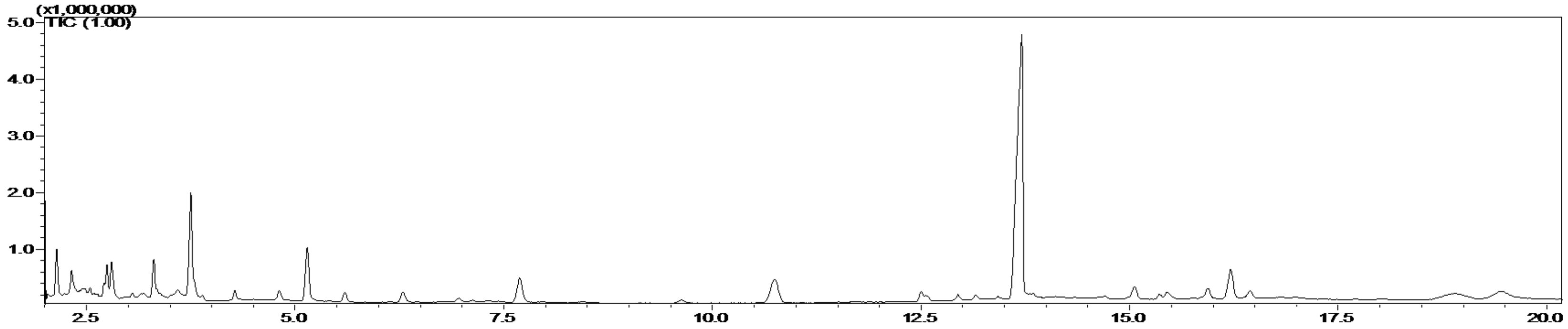

Figure S4. Representative chromatogram of the sprout extract of the original variety and the selected 10 mutant lines of wheat which were cultivated in a growth camber exposed to a blue LED light.

Wheat sprout samples	Gas chromatogram
WS57<br>PlotCW1027	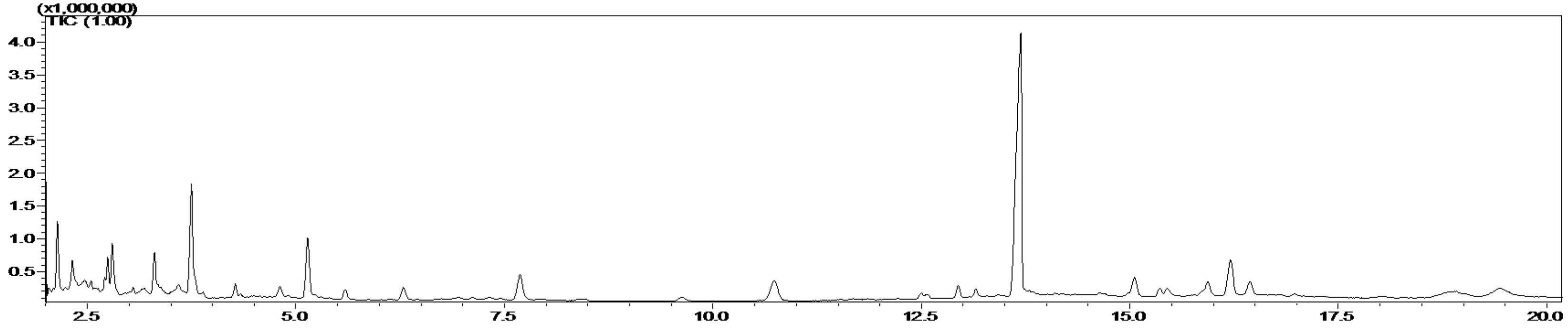
WS69<br>Plot80	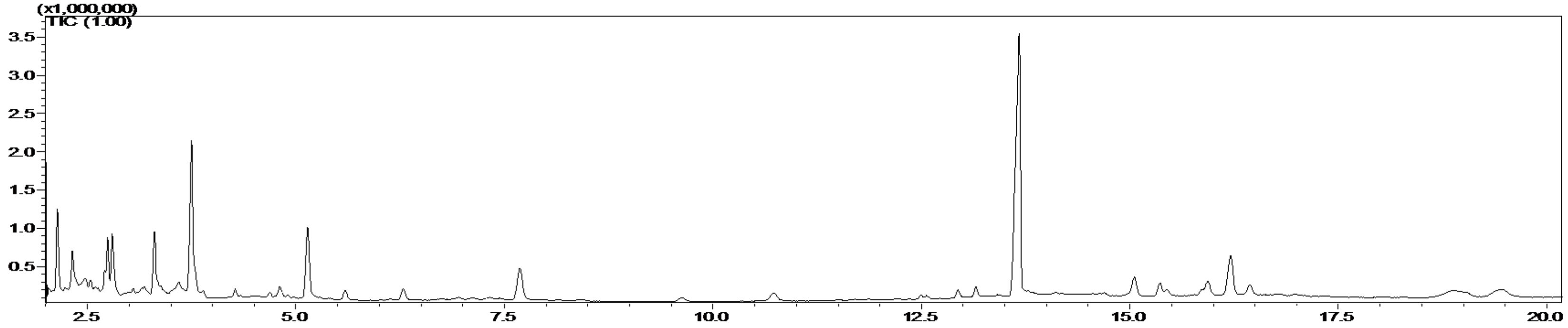
WS72<br>Plot83	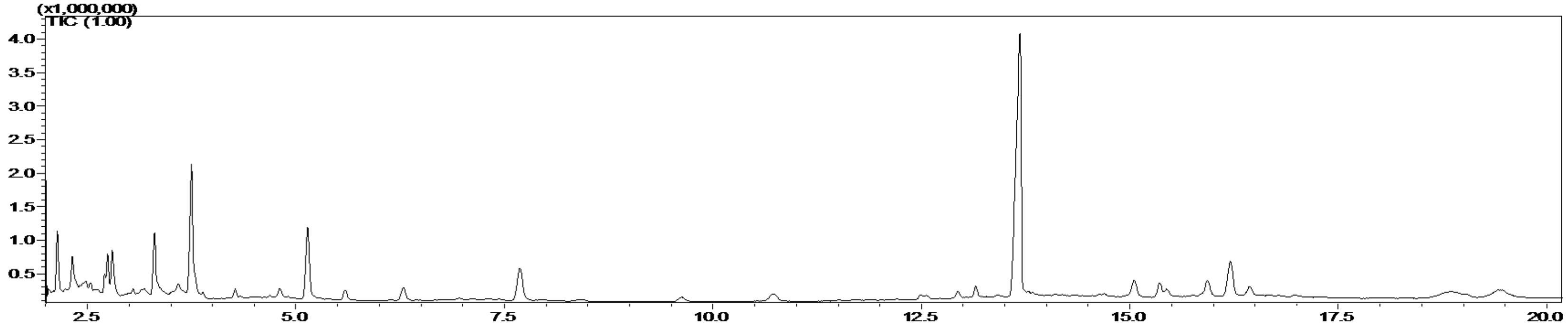

Figure S4. Representative chromatogram of the sprout extract of the original variety and the selected 10 mutant lines of wheat which were cultivated in a growth camber exposed to a blue LED light (*cont.*).

Wheat sprout samples	Gas chromatogram
WS74<br>Plot85	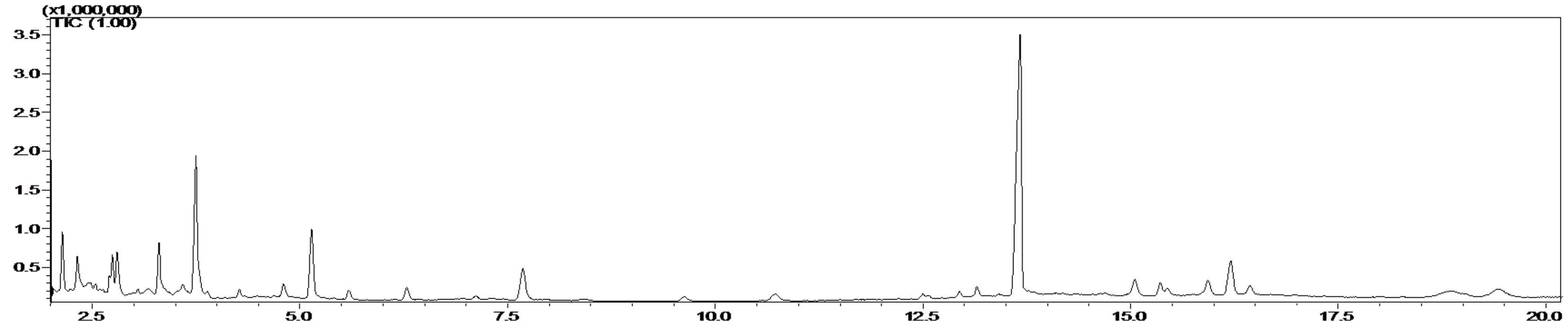
WS75<br>Plot86	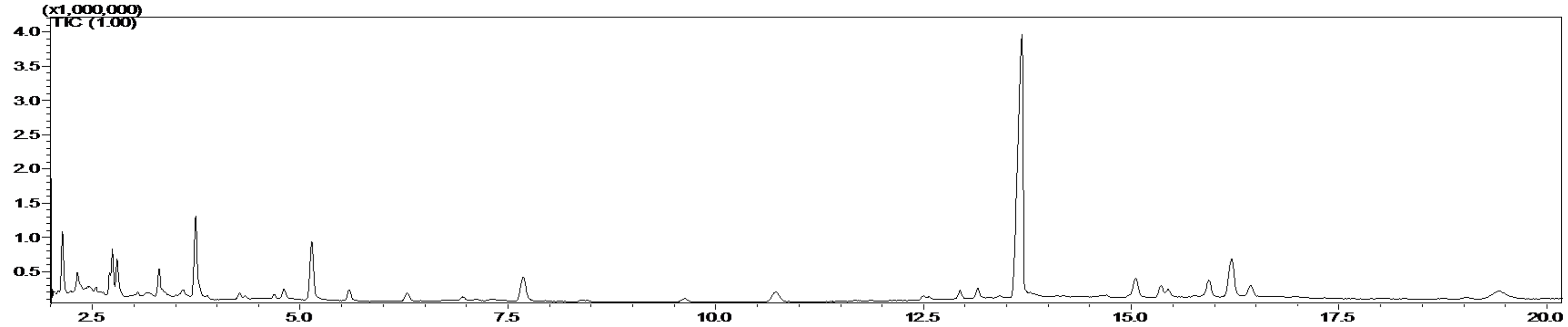
WS76<br>Plot87	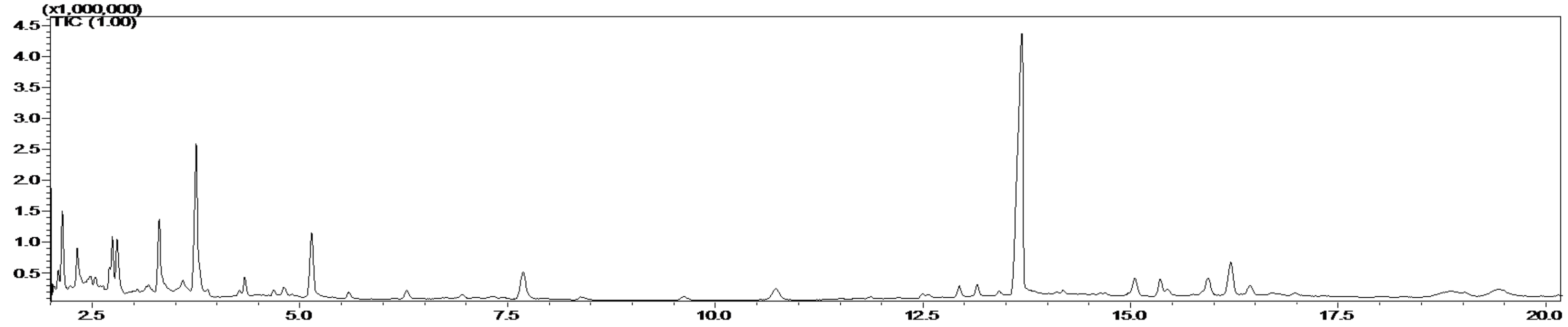

Figure S4. Representative chromatogram of the sprout extract of the original variety and the selected 10 mutant lines of wheat which were cultivated in a growth camber exposed to a blue LED light (*cont.*).

Wheat sprout samples	Gas chromatogram
WS78<br>Plot89	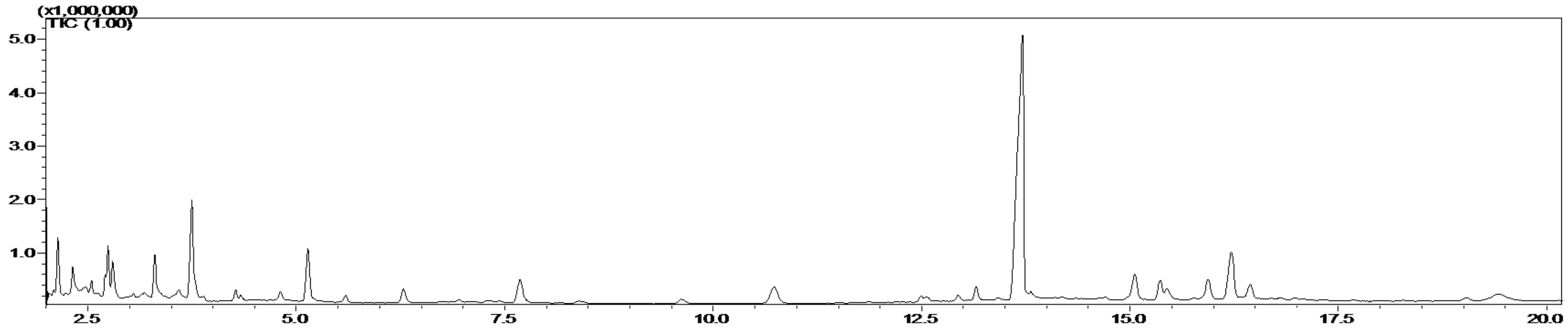
WS81<br>Plot92	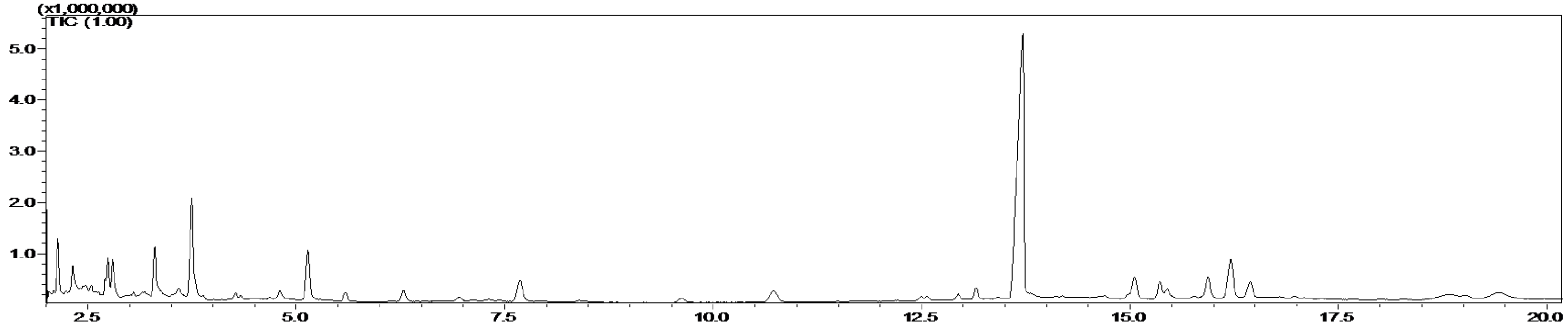

Figure S4. Representative chromatogram of the sprout extract of the original variety and the selected 10 mutant lines of wheat which were cultivated in a growth camber exposed to a blue LED light (*cont.*).

Wheat sprout samples	Gas chromatogram
WS01<br>Plot18	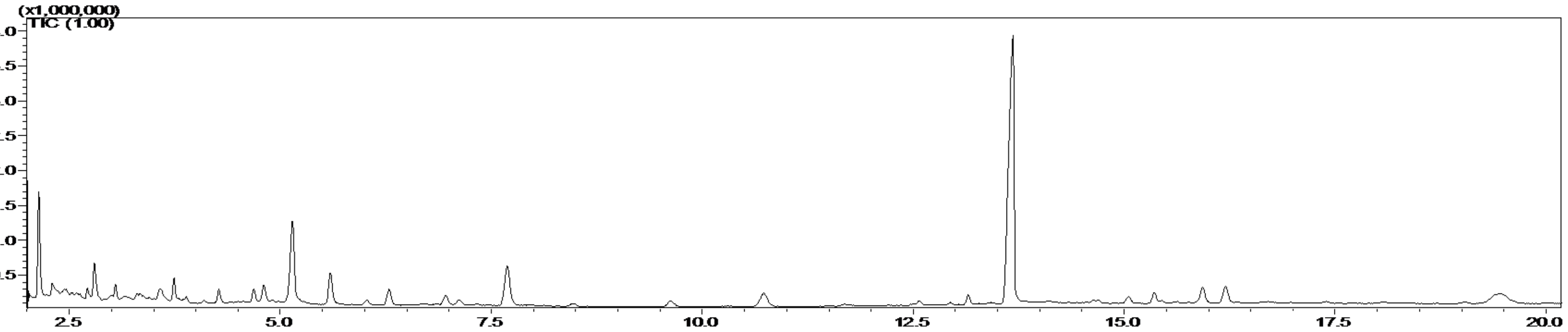
WS37<br>Plot46	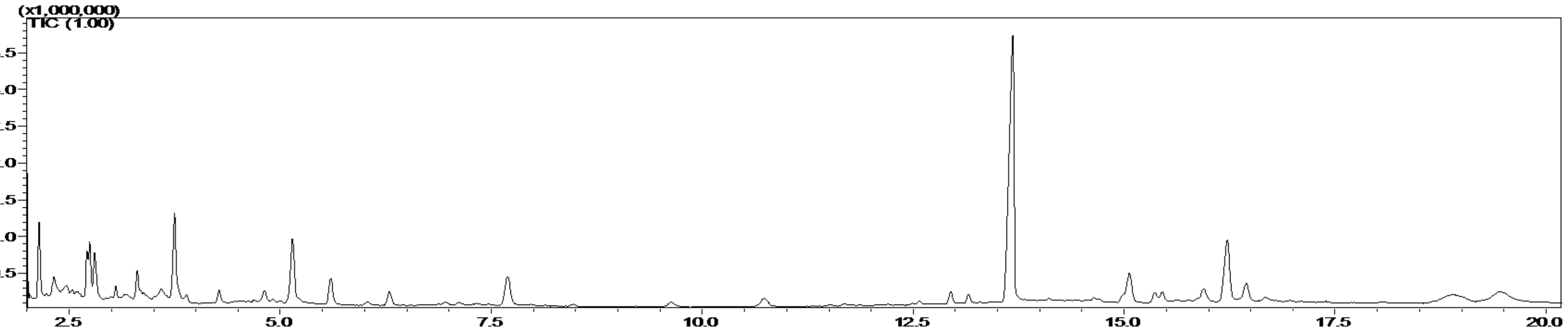
WS49<br>PlotCW263	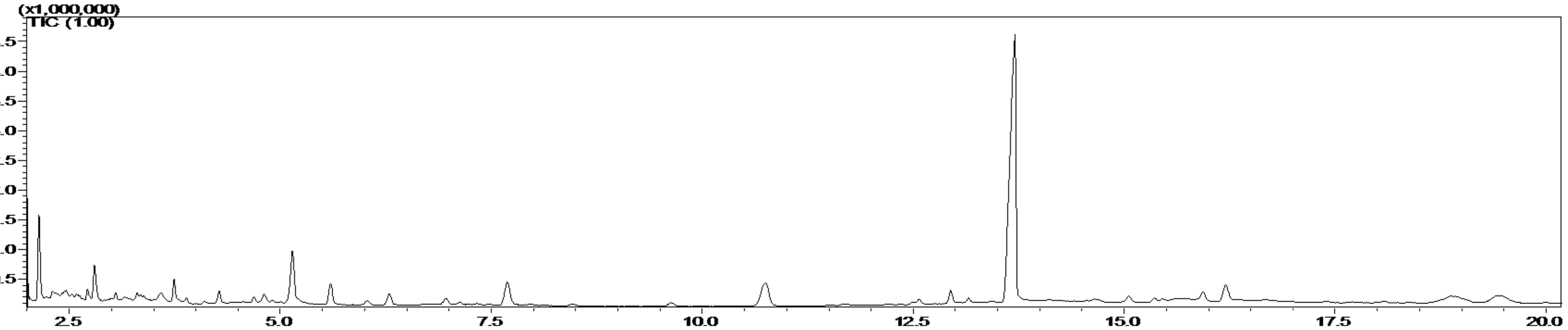

Figure S5. Representative chromatogram of the sprout extract of the original variety and the selected 10 mutant lines of wheat which were cultivated in a growth camber exposed to a green LED light.

Wheat sprout samples	Gas chromatogram
WS57<br>PlotCW1027	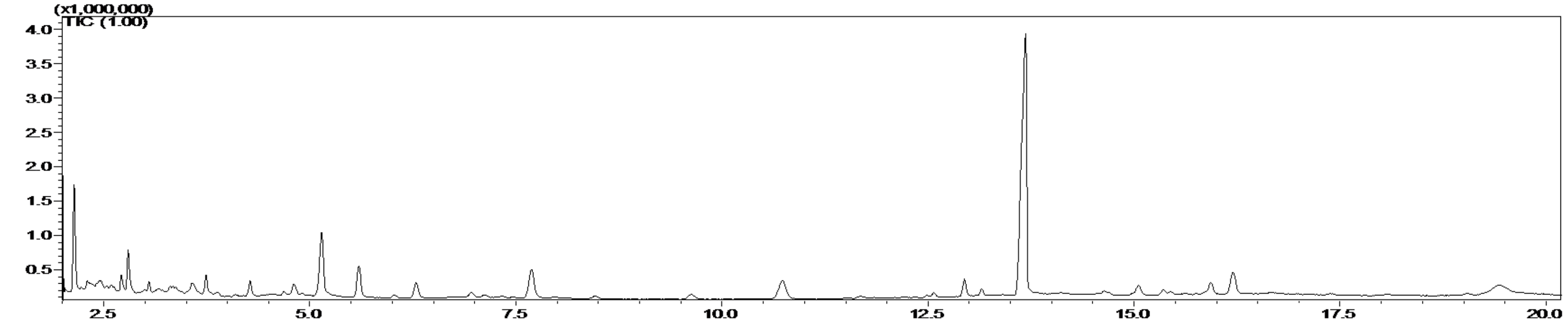
WS69<br>Plot80	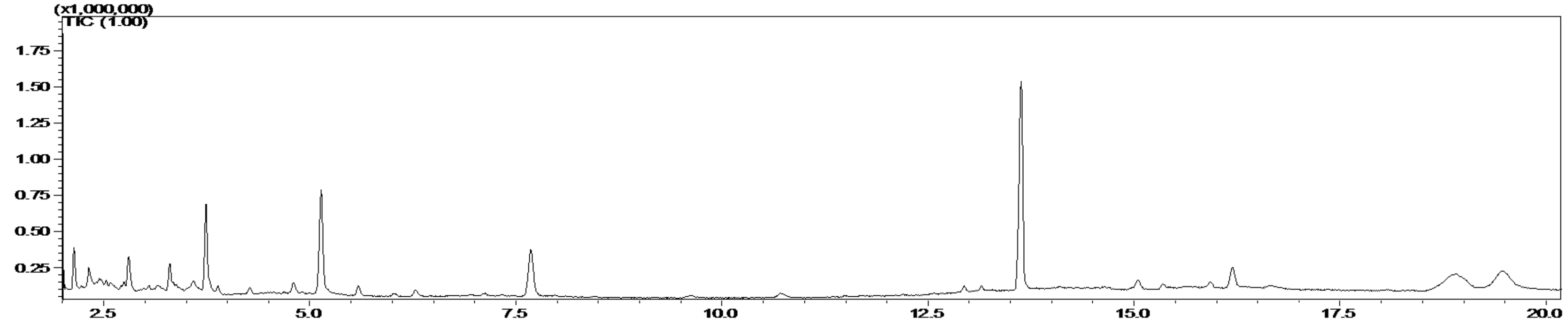
WS72<br>Plot83	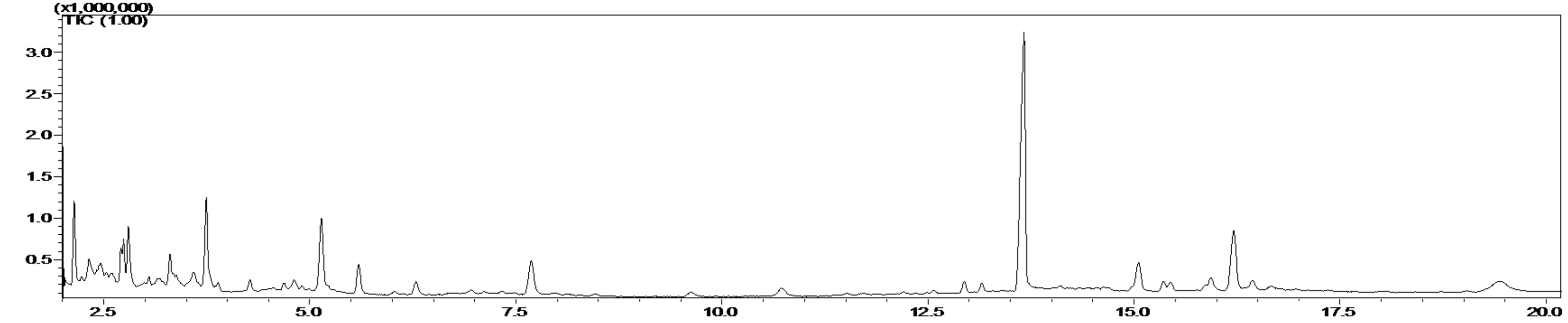

Figure S5. Representative chromatogram of the sprout extract of the original variety and the selected 10 mutant lines of wheat which were cultivated in a growth camber exposed to a green LED light (*cont.*).

<table>
<tr><th>Wheat sprout samples</th><th>Gas chromatogram</th></tr>
<tr><td>WS74
Plot85</td><td>
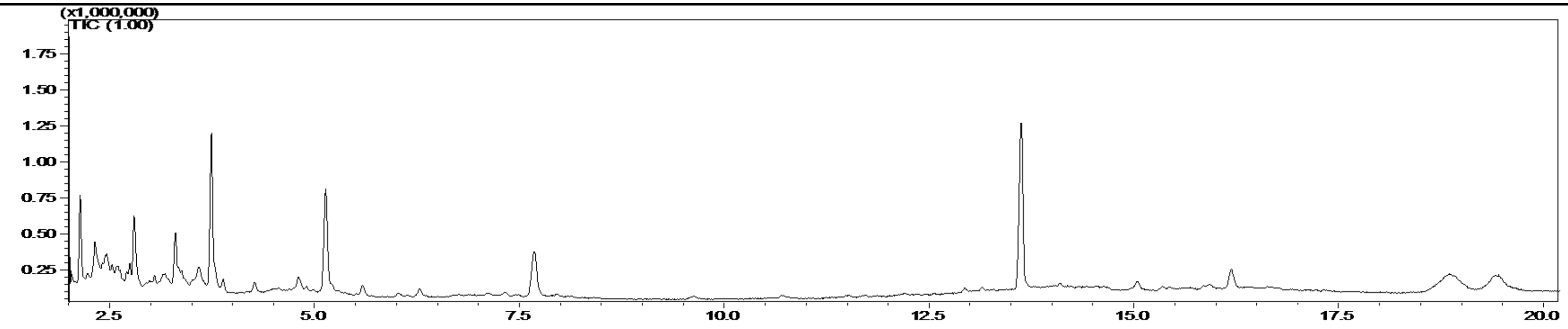

</td></tr>
<tr><td>WS75
Plot86</td><td>
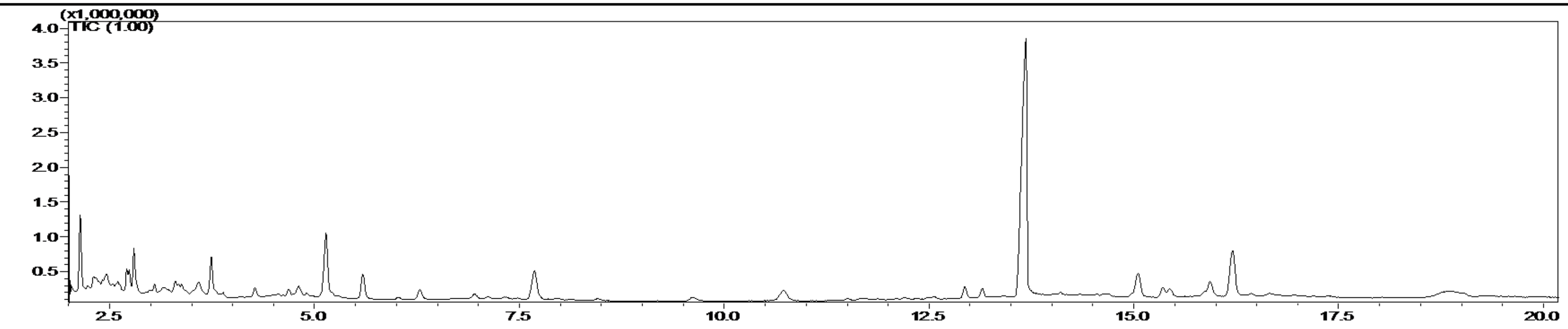

</td></tr>
<tr><td>WS76
Plot87</td><td>
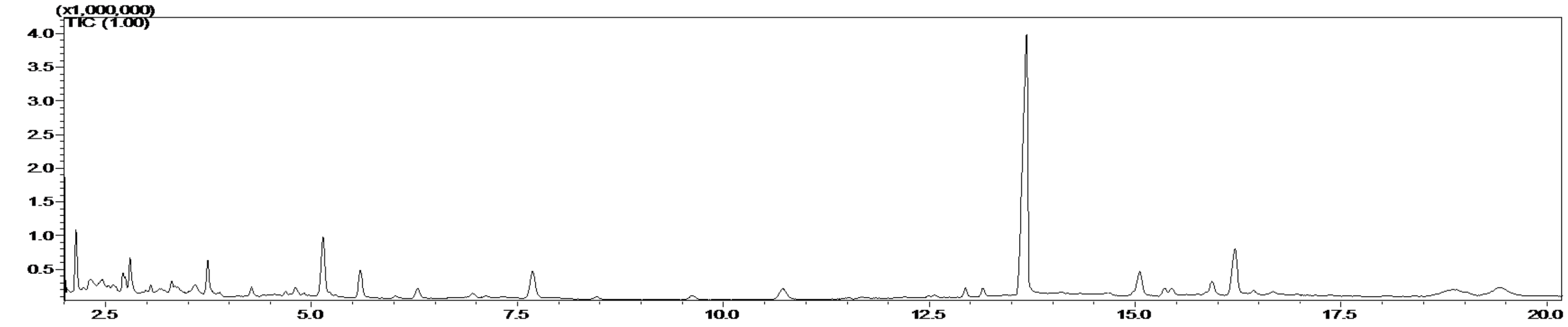

</td></tr>
</table>

Figure S5. Representative chromatogram of the sprout extract of the original variety and the selected 10 mutant lines of wheat which were cultivated in a growth camber exposed to a green LED light (*cont.*).

Wheat sprout samples	Gas chromatogram
WS78<br>Plot89	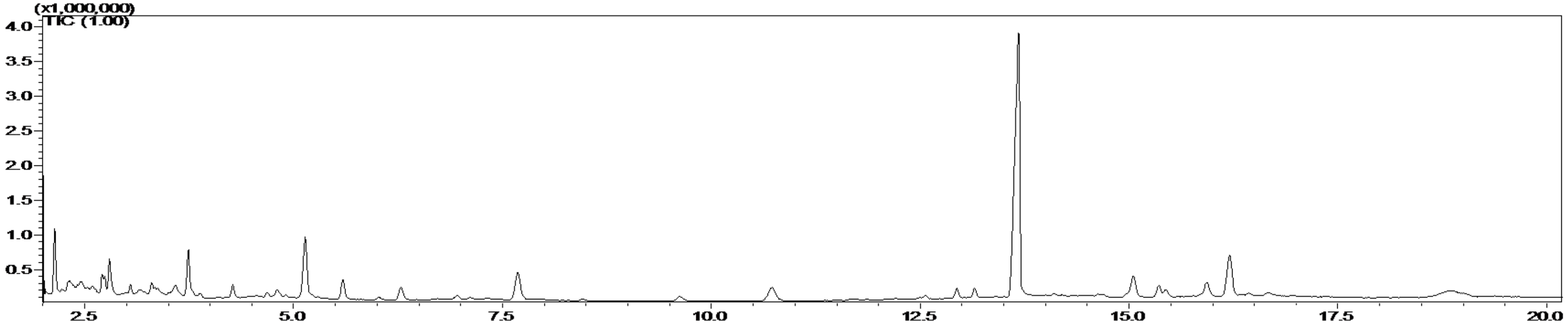
WS81<br>Plot92	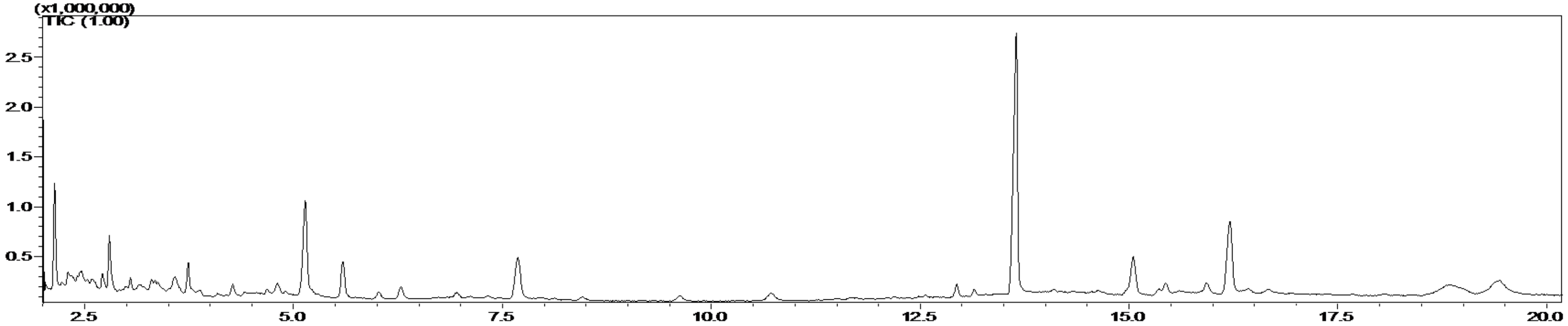

Figure S5. Representative chromatogram of the sprout extract of the original variety and the selected 10 mutant lines of wheat which were cultivated in a growth camber exposed to a green LED light (*cont.*).

Wheat sprout samples	Gas chromatogram
WS01<br>Plot18	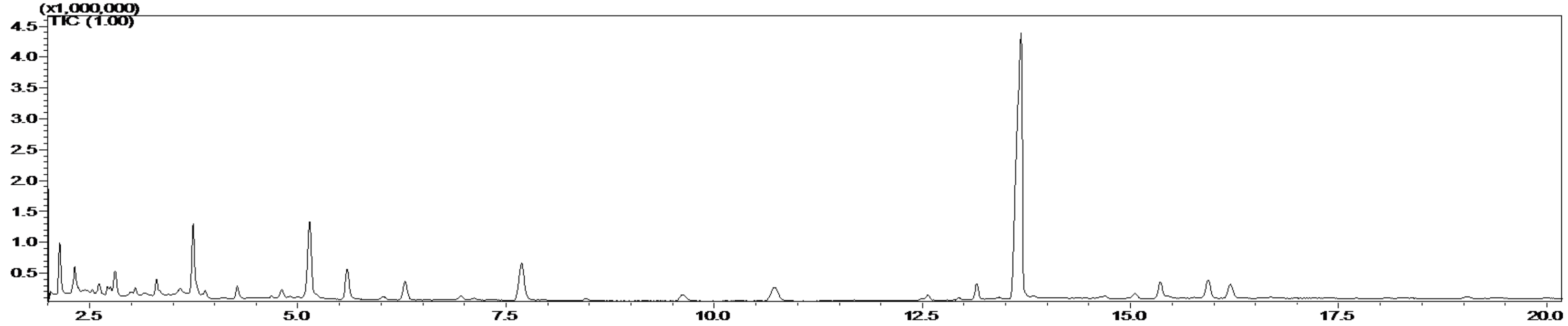
WS37<br>Plot46	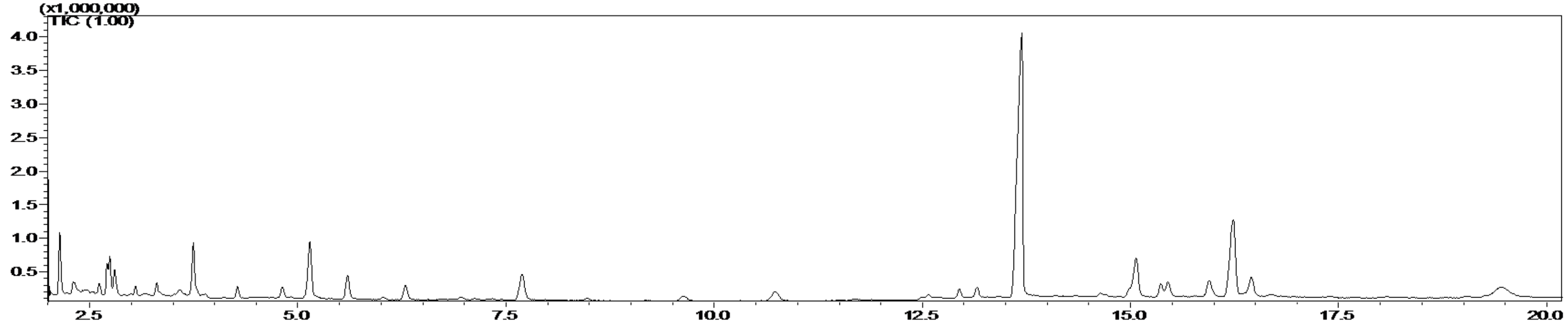
WS49<br>PlotCW263	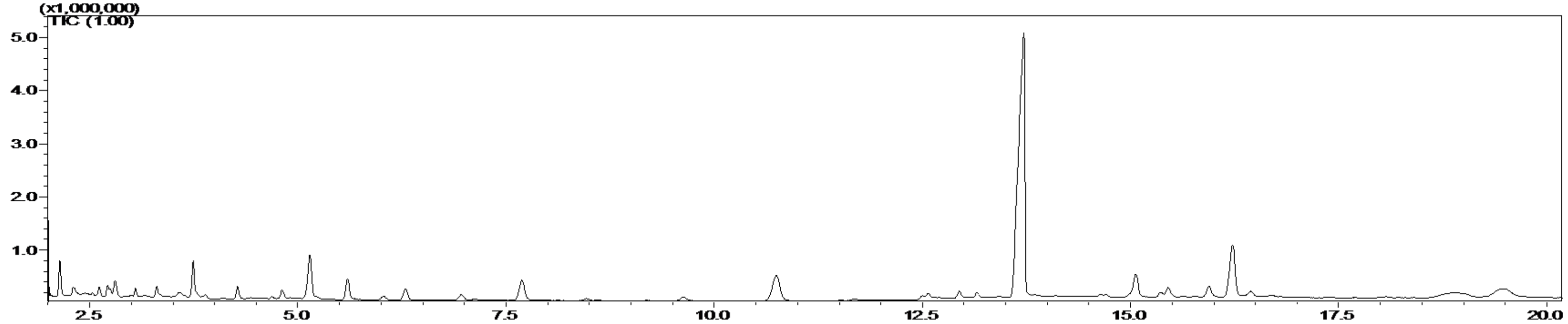

Figure S6. Representative chromatogram of the sprout extract of the original variety and the selected 10 mutant lines of wheat which were cultivated in a growth camber exposed to a red LED light.

Wheat sprout samples	Gas chromatogram
WS57<br>PlotCW1027	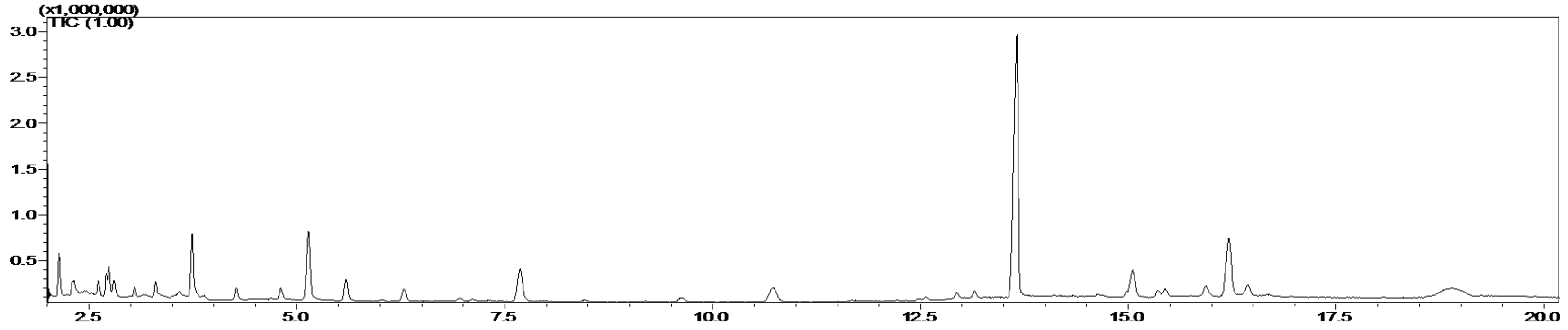
WS69<br>Plot80	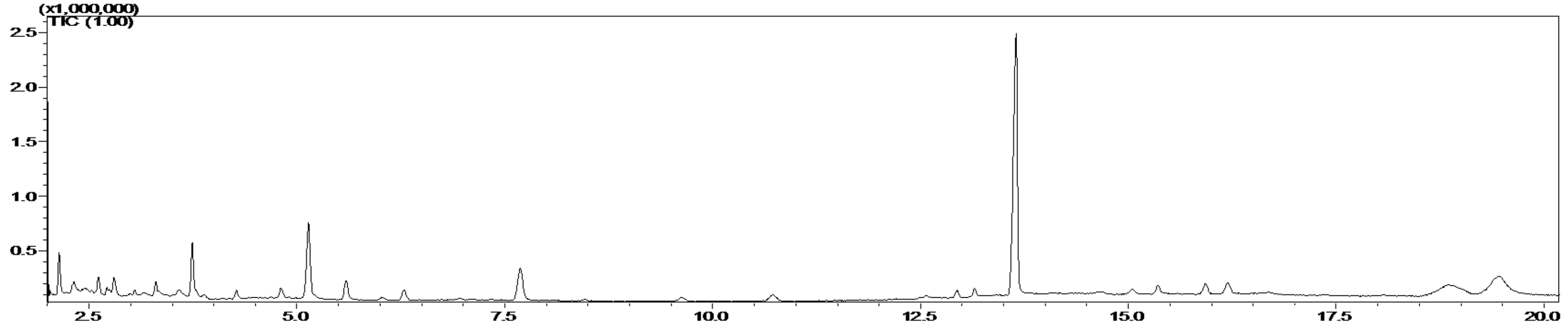
WS72<br>Plot83	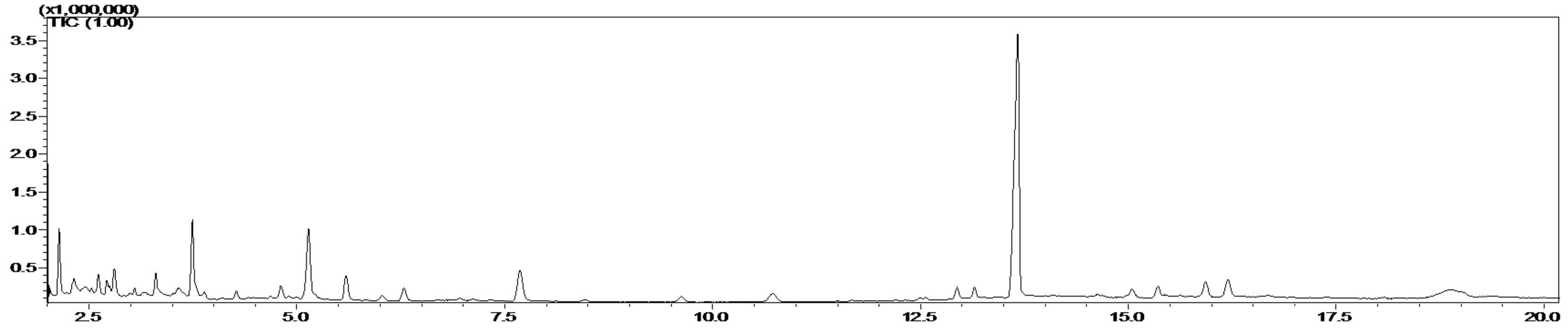

Figure S6. Representative chromatogram of the sprout extract of the original variety and the selected 10 mutant lines of wheat which were cultivated in a growth camber exposed to a red LED light (*cont.*).

Wheat sprout samples	Gas chromatogram
WS74<br>Plot85	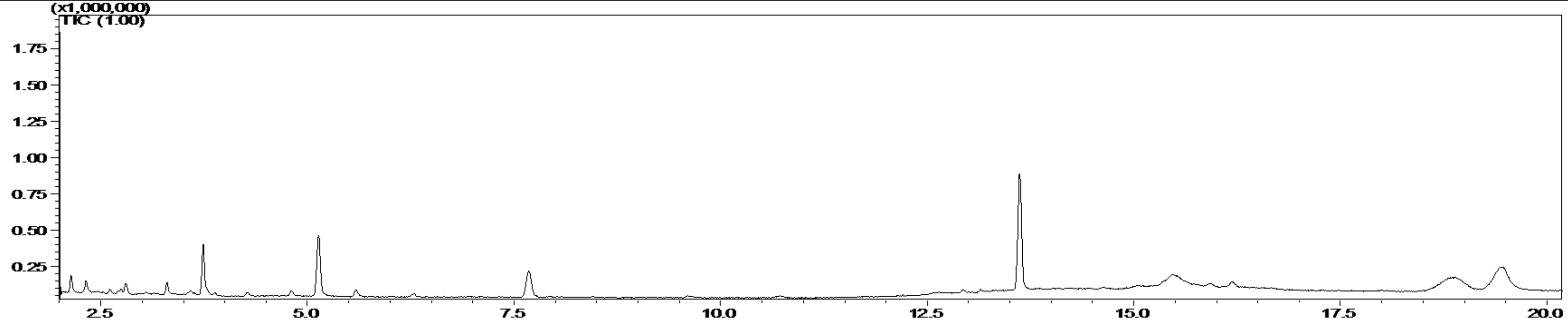
WS75<br>Plot86	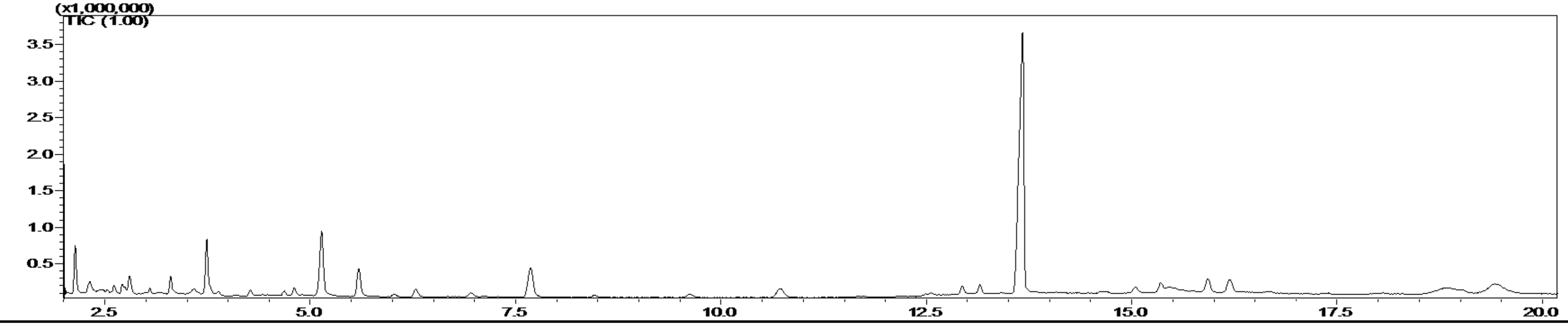
WS76<br>Plot87	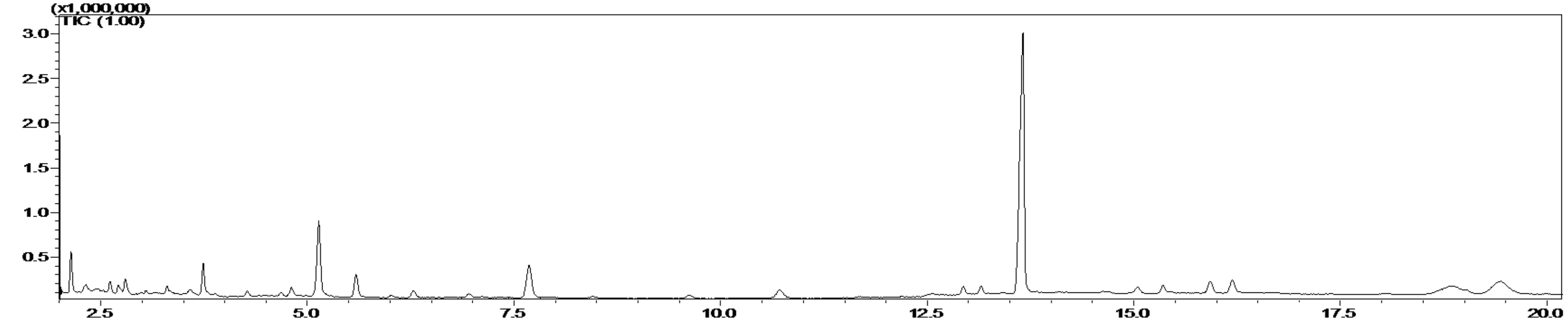

Figure S6. Representative chromatogram of the sprout extract of the original variety and the selected 10 mutant lines of wheat which were cultivated in a growth camber exposed to a red LED light (*cont.*).

Wheat sprout samples	Gas chromatogram
WS78<br>Plot89	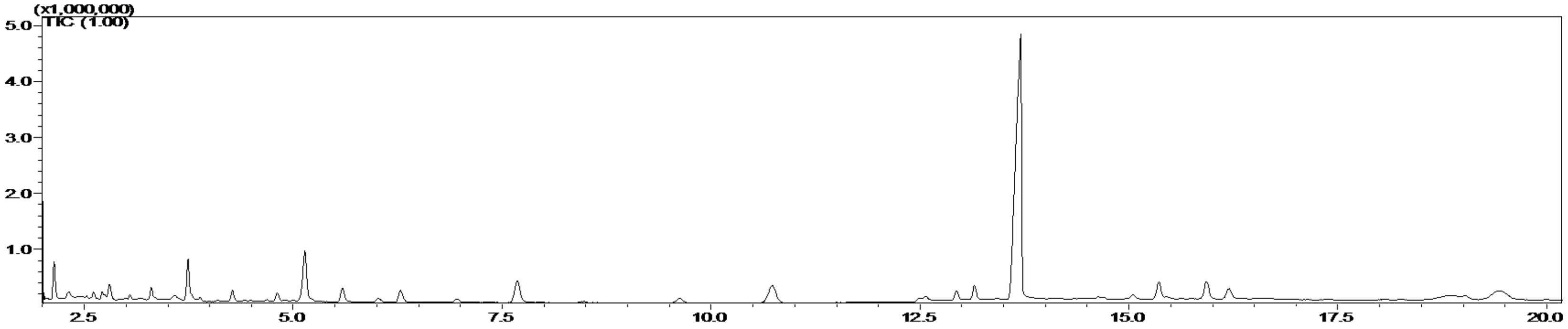
WS81<br>Plot92	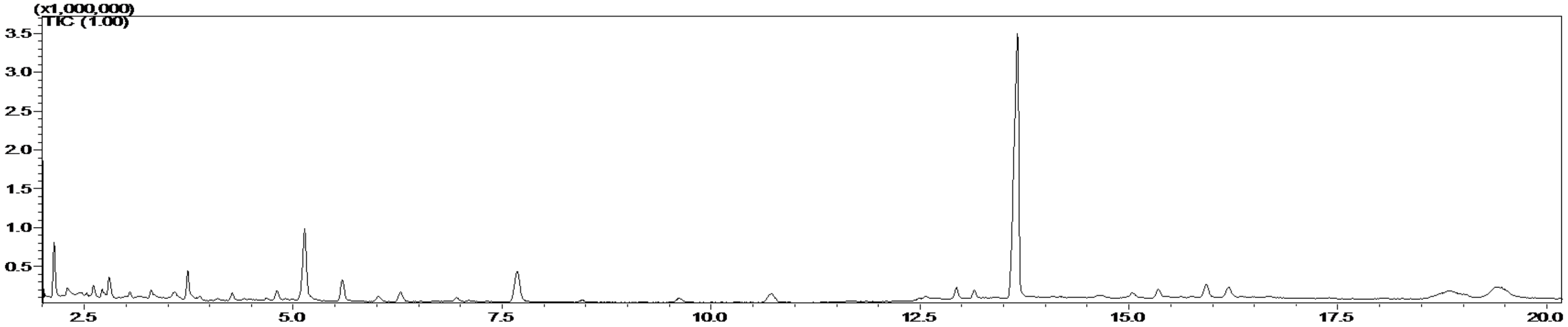

Figure S6. Representative chromatogram of the sprout extract of the original variety and the selected 10 mutant lines of wheat which were cultivated in a growth camber exposed to a red LED light (*cont.*).
